# Red Light Controls Adventitious Root Regeneration by Modulating Hormone Homeostasis in *Picea abies* Seedlings

**DOI:** 10.3389/fpls.2020.586140

**Published:** 2020-09-09

**Authors:** Sanaria Alallaq, Alok Ranjan, Federica Brunoni, Ondřej Novák, Abdellah Lakehal, Catherine Bellini

**Affiliations:** ^1^ Umeå Plant Science Centre, Department of Plant Physiology, Umeå University, Umeå, Sweden; ^2^ Department of Biology, College of Science for Women, Baghdad University, Baghdad, Iraq; ^3^ Umeå Plant Science Centre, Department of Forest Genetics and Physiology, Swedish Agriculture University, Umea, Sweden; ^4^ Laboratory of Growth Regulators, Faculty of Science, Palacký University and Institute of Experimental Botany, The Czech Academy of Sciences, Olomouc, Czechia; ^5^ Institut Jean-Pierre Bourgin, INRAE, AgroParisTech, Université Paris-Saclay, Versailles, France

**Keywords:** adventitious roots, conifers, *Picea abies*, auxin, cytokinins, jasmonate, red light, root development

## Abstract

Vegetative propagation relies on the capacity of plants to regenerate *de novo* adventitious roots (ARs), a quantitative trait controlled by the interaction of endogenous factors, such as hormones and environmental cues among which light plays a central role. However, the physiological and molecular components mediating light cues during AR initiation (ARI) remain largely elusive. Here, we explored the role of red light (RL) on ARI in de-rooted Norway spruce seedlings. We combined investigation of hormone metabolism and gene expression analysis to identify potential signaling pathways. We also performed extensive anatomical characterization to investigate ARI at the cellular level. We showed that in contrast to white light, red light promoted ARI likely by reducing jasmonate (JA) and JA-isoleucine biosynthesis and repressing the accumulation of isopentyl-adenine-type cytokinins. We demonstrated that exogenously applied JA and/or CK inhibit ARI in a dose-dependent manner and found that they possibly act in the same pathway. The negative effect of JA on ARI was confirmed at the histological level. We showed that JA represses the early events of ARI. In conclusion, RL promotes ARI by repressing the accumulation of the wound-induced phytohormones JA and CK.

## Introduction

Vegetative **(**or clonal**)** propagation through stem cuttings is widely used in breeding programs for propagation of elite genotypes. Successful clonal propagation requires *de novo* regeneration of adventitious roots (ARs), a process governed by a multitude of developmentally programmed and environmentally induced cues including wounding, light, and nutrient availability (Bellini et al., 2014; Steffens and Rasmussen, 2016). Because AR regeneration is a quantitative trait, the rooting capacity of cuttings varies among individuals within species, populations, or even clones (Abarca and Díaz-Sala, 2009). Phytohormones, mainly auxin, jasmonate (JA), cytokinins (CKs), ethylene, and abscisic acid (ABA), play major roles in AR formation. It has been shown that there is interplay among these phytohormones in complex networks and loops that ensure proper AR development (Lakehal and Bellini, 2019). For example, in *Arabidopsis thaliana*, it has been shown that auxin signaling controls AR formation by modulating JA homeostasis in the hypocotyl (Gutierrez et al., 2012; Lakehal et al., 2019a) and that JA cooperates with CKs to repress AR formation (Lakehal et al., 2020a). Moreover, JA induces the expression of *DIOXYGENASE FOR AUXIN OXIDATION 1 (DAO1)* in a *CORNATINE INSENSTIVE1*(*COI1*)-dependent way. DAO1 is a key enzyme catalyzing the irreversible degradation of auxin, to ensure robust crosstalk between auxin and JA signaling pathways (Lakehal et al., 2019b). However, the extent to which these complex molecular networks are evolutionarily conserved among plant species is poorly understood, especially for trees. Although it remains difficult to address the molecular mechanisms controlling AR formation in perennial species, the rapid development of sequencing technologies giving access to most genome and transcriptome sequences now offers the possibility of further exploring the degree of evolutionary conservation or divergence of the known molecular networks.

Light is one of the most important external cues controlling plant growth and development. It is perceived by highly sophisticated sensing machinery which mediates a plethora of physiological and developmental processes, *inter alia* photosynthesis, photomorphogenesis, flowering time, and tropisms (de Wit et al., 2016). Depending on light intensity and spectral quality, the cues perceived trigger specific downstream transcriptional signatures enabling a plant to adjust its growth and development as well as its response to the environment. Phytohormones play central roles in integrating and translating cues into developmentally coherent outputs and responses (de Wit et al., 2016). In particular, light intensity and spectral quality have long been manipulated as tools in vegetative propagation practices in order to stimulate the rooting ability of recalcitrant species. For example, treating donor plants of difficult-to-root *Eucalyptus globulus* species with far-red-enriched light stimulated the rooting capacity of microcuttings (Ruedell et al., 2015). This stimulation was possibly due to upregulation of the expression of *AUXIN RESPONSE FACTOR6-like (ARF6-like)* and *ARF8-like* genes at the cutting site by far-red light. These transcriptional regulators have been shown to promote ARI in Arabidopsis hypocotyls (Gutierrez et al., 2009; Gutierrez et al., 2012) and in *Populus* stem cuttings (Cai et al., 2019). Far-red-enriched light also induced the expression of a putative indole 3-acetic acid (IAA) biosynthetic gene, *YUCCA3-like*, and two putative auxin efflux carriers genes, *PIN-FORMED 1-like (PIN1-like).* and *PIN-FORMED 2-like (PIN2-like)*, in *Eucalyptus globulus* (Ruedell et al., 2015). These data suggest that far-red-enriched light might act on the auxin biosynthesis, transport, and/or signaling pathways to stimulate AR formation in *Eucalyptus globulus.* Keeping leafy stem cuttings of *Petunia hybrida* (Klopotek et al., 2016) or *Dianthus caryophyllus* (Agulló-Antón et al., 2011) in dark conditions prior to light exposure improved their rooting capacity. The effect of light on Norway spruce (*Picea abies*) growth and morphogenesis has also been documented (Strömquist and Eliasson, 1979; Mortensen and Sandvik, 1988; Bollmark and Eliasson, 1990; Mølmann et al., 2006; Burescu et al., 2015; OuYang et al., 2015), and it has been shown that high-irradiance white light inhibited AR in Norway spruce stem cuttings (Strömquist and Eliasson, 1979).

Despite the importance of light in regulating AR formation, the physiological and molecular mechanisms involved are still unknown. Here, we used de-rooted Norway spruce seedlings as a model system to explore the role of light in the control of AR initiation in conifers. We found that constant red light promotes this process, possibly through downregulation of JA signaling and repression of cytokinin accumulation compared to constant white light conditions. It appears that the cooperative role of JA and CK signaling in the repression of ARI is evolutionarily conserved, as it represses very early events in the ARI process in Norway spruce hypocotyls.

## Materials and Methods

### Plant Material

Commercial seeds from Norway spruce (*Picea abies* L. Karst) were provided by Svenska Skogsplantor (https://www.skogsplantor.se/; Sweden) and kept at 4 **°**C prior to use. The seeds were soaked in tap water for 24 h, in the dark at 4 **°**C. They were then sown in fine wet vermiculite as described in Ricci et al. (2008) and allowed to germinate and grow in a growth chamber. The seedlings were watered twice a week with tap water and left to grow for 3 weeks (**Figure S1A**). The growth conditions were as follow: 16 h light with 75 μmol/m^2^/s (400 to 700 nm, **Figure S2A**); 8 h dark; 20°C day temperature and 18°C night temperature. The white light was provided by 36W/840 Super 80 Cool White TL-D tubes (Philips, https://www.philips.se/).

### Test of Effects of Different Light Conditions on Adventitious Rooting of De-Rooted Seedlings

Twenty-one days after sowing, the seedling hypocotyls were severed 2 cm below the apex (**Figure S1B**) and placed in 24 ml vials filled with distilled water (**Figure S1C**). Three vials containing 5 seedlings each, constituting technical replicates, were prepared.

In order to test different white light conditions, vials containing de-rooted seedlings were kept in either long-day conditions in the growth chamber as described above (**Figure S2A**) or in a Percival growth cabinet equipped with cool white fluorescent tubes (F17T8/TL741, 17 Watt; Philips U.S.A: Percival Scientific, Perry Iowa, USA) (**Figure S2B**) or in a Percival plant growth cabinet (E-30NL/*floraLED*) equipped with six CLF *floraLED* modules (CLF PlantClimatics GmbH; Emersacker, Germany) providing white, or red light (**Figures S2C, D**). Under these latter conditions the light was constant and referred to as constant White Light (cWL), or constant Red Light (cRL), and the temperature was constant and set at 20°C.

The cRL (660 nm) was set at an intensity of 9 μmol/m^2^/s (**Figure S2D**).

The vials were refilled daily with distilled water to replace the water lost by evapotranspiration. The number of roots per cutting and the number of rooted cuttings were monitored for 30 days after cutting (DAC) and all the experiments were repeated at least three times.

### Hormone Treatments

#### Auxin Treatment

Five hypocotyl cuttings per vial, with three technical replicates, were cultured in 24 ml of distilled water supplemented with indole butyric acid (IBA), indole acetic acid (IAA) or 1-Naphthalene Acetic Acid (NAA) at final concentrations of 1, 5, or 10 μM, or without supplementation as mock treatment.

#### Jasmonate (JA) Treatment

Five hypocotyl cuttings per vial, with three technical replicates, were cultured in 24 ml of distilled water supplemented with JA at final concentrations of 2 μM, 10 μM or 20 μM, or without supplementation as mock treatment.

#### Cytokinin Treatment

Five hypocotyl cuttings per vial, with three technical replicates, were cultured in 24 ml of distilled water supplemented with 6-benzylaminopurine (BAP) at a final concentration of 0.1 μM or 0.01 μM, or without supplementation.

BAP was also used in combination with 1 μM IBA, 2 μM or 5 μM JA.

For all the hormones concentrated stock solutions were prepared with ethanol 90%. For hormone treatment few μl of stock solution were added to distilled water, and the same amount of ethanol was used for mock. Final pH of the mock and hormone solution was 5.2.

In all these experiments vials were covered with parafilm in order to limit the evaporation of hormone solutions during the rooting period**;** small holes allowed the cuttings to be inserted into the vials.

### Histology

Five mm sections of hypocotyl cuttings were dissected from 3-week-old seedlings at T0, just before transferring the cuttings to rooting conditions, then 3, 5, 10, 13, and 15 days after cutting. Similar material was taken from de-rooted seedlings treated with 1 μM IBA or 20 μM JA. Hypocotyl samples were vacuum infiltrated with a fixation medium (10 ml of formaldehyde 37%, 5 ml of acetic acid 5%, 50 ml of ethanol 100% and 35 ml of H_2_O) for 20 s and left for 24 h at room temperature. The samples were then washed in ethanol (70%) for 10 min and transferred into EtOH 70% until required for use. Samples were then gradually dehydrated in an ethanol series (80%, 90%, 96% for 2 h each and 100% overnight at room temperature). The 100% EtOH was gradually replaced by HistoChoice tissue fixative (VWR Life, https://us.vwr.com/) in three steps of 1:3, 1:1, 3:1 (EtOH: HistoChoice ratio) and two 1 h steps with pure HistoChoice. The HistoChoice fixative was gradually replaced with Paraplast Plus for tissue embedding (Sigma-Aldrich, https://www.sigmaaldrich.com/), over 6 days. Serial sections (10 μm) were obtained with a Zeiss HM 350 rotary microtome (https://www.zeiss.com/). Longitudinal and transverse sections were stained with safranin (Sigma-Aldrich, https://www.sigmaaldrich.com/) and alcian blue (Sigma-Aldrich, https://www.sigmaaldrich.com/) in a ratio of 1:2; using methods from (Hamann et al., 2011).

### Sources of Sequence Data and Phylogenetic Tree Construction

The *AtMYC2* (At1g32640), *AtJAZ3* (At3g17860), *AtJAZ10* (At5g13220), *AtAOC1* (At3g25760), and *AtCOI1* (At2g39940) *Arabidopsis thaliana* gene coding sequences (CDS) from TAIR (https://www.arabidopsis.org) were used as queries in BLASTN searches for CDS predicted with high confidence from *P. abies* (http://congenie.org/; Nystedt et al., 2013) to identify potential orthologs in the *Picea abies* genome. All sequence information is provided in **Figure S4**. To find the putative Arabidopsis orthologs in *Picea abies*, a BLAST alignment of the coding sequences of selected genes was generated using “Genome tools” from the Congenie database with default settings. Subsequently, the sequences were used to construct phylogenetic trees with the tools available at “http://www.phylogeny.fr”. The phylogenetic analysis was based on the neighbor-joining method (with 1000 bootstrap replicates). The putative orthologs were named according to the Congenie database.

### Primer Design, Efficiency, and qRT-PCR Analysis

Candidate genes were identified by BLASTN searches against the spruce database (http://congenie.org) using Arabidopsis protein coding nucleotide sequences. The Norway spruce genes that showed the highest sequence similarity with Arabidopsis genes were further considered for primer design and gene expression analysis. Primers for the selected candidate genes and reference genes to be used for qRT-PCR were designed using Primer3web version 4.1.0 (http://primer3.ut.ee); the primer sequences are listed in **Table S1**. Specificity of the primers was analyzed by Sanger sequencing of amplified PCR products and cDNA was used as a template for PCR amplification from each primer pair. For each primer pair, PCR reaction efficiency estimates were calculated from a standard curve generated from a serial dilution of cDNA. Based on the average cycle threshold (Ct) values for each five-fold dilution, a standard curve was generated using linear regression. PCR efficiency (E) was derived from the equation: Efficiency % = (10 (−1/slope) − 1) ×100%. PCR conditions were as follows: 95°C for 1 min, 40 cycles of 95°C for 15 s, 60°C for 30 s, and 72°C for 30 s. Finally, dissociation curves were generated by increasing the temperature from 65 to 95°C, stepwise by 0.3°C every 10 s. For gene expression analysis by quantitative PCR (qPCR), total RNA was isolated from the base of spruce hypocotyls (5 mm long from 36 to 38 hypocotyl cuttings) using a Spectrum™ Plant Total RNA Kit (Sigma-Aldrich; https://www.sigmaaldrich.com/). cDNA was synthesized using an iScript™ cDNA Synthesis Kit (Bio‐Rad; https://www.bio-rad.com/en-se/) following DNase treatment. Expression was quantified by the LightCycler® 480 System (Roche, Basel, Switzerland) using LightCycler® 480 SYBR Green I Master (Roche, Basel, Switzerland). For each sample, three technical replicates from two independent biological replicates were analyzed. The ΔΔ^CT^ method was used to measure the relative amount of transcript from each gene. Expression values were normalized to the expression of two reference genes, *PaEIF4A1* and *PaUBC28*, and calibrated with the control value arbitrarily set to 1.

In order to check whether expression of the selected putative orthologs *PaMYC2-like*, *PaAOC1-like*, *PaJAZ3-like*, and *PaJAZ10-like* could be induced by jasmonate, 3-week-old whole Norway spruce seedlings were treated with 50 µM of JA or mock treatment for 3 h. Total RNA was prepared and qPCR experiments performed as described above.

### Hormone Profiling Experiments

For hormone quantification, 5 mm hypocotyl segments were dissected at the base of de-rooted hypocotyls 6, 24, 48, 72, and 120 h after they had been transferred to cWL, cRL, in order to provide an average of 20 mg of fresh weight.

Quantification of cytokinin metabolites was performed according to the method described by (Svačinová et al., 2012), including modifications described by (Antoniadi et al., 2015). Samples (20 mg fresh weight, FW) were homogenized and extracted in 1 ml of modified Bieleski buffer (60% MeOH, 10% HCOOH, and 30% H_2_O) together with a cocktail of stable isotope-labeled internal standards (0.25 pmol of CK bases added per sample). The extracts were applied to an Oasis MCX column (30 mg/1 ml, Waters) conditioned with 1 ml each of 100% MeOH and H_2_O, equilibrated sequentially with 1 ml of 50% (v/v) nitric acid, 1 ml of H_2_O, and 1 ml of 1M HCOOH, and washed with 1 ml of 1M HCOOH and 1 ml 100% MeOH. Analytes were then eluted in two steps using 1 ml of 0.35M NH_4_OH aqueous solution and 2 ml of 0.35M NH_4_OH in 60% (v/v) MeOH. The eluates were then evaporated to dryness *in vacuo* and stored at −20°C. Cytokinin levels were determined by ultra-high performance liquid chromatography-electrospray tandem mass spectrometry (UHPLC-MS/MS) using stable isotope-labeled internal standards as a reference (Rittenberg and Foster, 1940). Separation was performed on an Acquity UPLC® System (Waters, Milford, MA, USA) equipped with an Acquity UPLC BEH Shield RP18 column (150 mm × 2.1 mm, 1.7 μm; Waters), and the effluent was introduced into the electrospray ion source of a triple quadrupole mass spectrometer Xevo™ TQ-S MS (Waters). Six independent biological replicates were performed.

Endogenous levels of free IAA, ABA, SA, and JA, as well as the conjugated form of JA, JA-Ile, in 20 mg of hypocotyls were determined according to the method described in (Floková et al., 2014). The phytohormones were extracted using an aqueous solution of methanol (10% MeOH/H_2_O, v/v). To validate the LC-MS method, a cocktail of stable isotope-labeled standards was added with the following composition: 5 pmol of [^13^C_6_]IAA, 10 pmol of [^2^H_6_]JA, [^2^H_2_]JA-Ile and 20 pmol of [^2^H_4_]SA (all from Olchemim Ltd, Czech Republic) per sample. The extracts were purified using Oasis HLB columns (30 mg/1 ml, Waters, Milford, MA, USA) and targeted analytes were eluted using 80% MeOH. Eluate containing neutral and acidic compounds was gently evaporated to dryness under a stream of nitrogen. Separation was performed on an Acquity UPLC® System (Waters, Milford, MA, USA) equipped with an Acquity UPLC BEH C18 column (100 × 2.1 mm, 1.7 μm; Waters, Milford, MA, USA), and the effluent was introduced into the electrospray ion source of a triple quadrupole mass spectrometer Xevo™ TQ-S MS (Waters, Milford, MA, USA).

### Statistical Analysis

For statistical analyses, the SPSS 20 software package was used (https://www.ibm.com/support/pages/spss-statistics-20). Student’s t-tests were performed to evaluate the effects of different light qualities on hormone contents. Repeated measures ANOVAs were used to test the effects of time and hormone concentrations under constant red light.

## Results

### Auxin Is Not Sufficient to Stimulate Adventitious Rooting in Norway Spruce Hypocotyl Cuttings Kept Under White Light

To investigate the role of light in adventitious root initiation (ARI) in Norway spruce, we used de-rooted Norway spruce seedlings as a model system. Three-week-old seedlings grown under white light condition were harvested and de-rooted as described in *Materials and* *Methods* (**Figure S1**). They were first kept in hormone free (HF) distilled water, under three different white light (WL) regimes as described in *Materials and* *Methods* (irradiance 69 to 75 µM/m^2^/s^−1^; **Figures S2A–C**). In these conditions no AR developed at the base of the hypocotyls regardless of the photoperiod or the source of white light. Since we obtained the same results in all WL conditions, we continued to use constant WL (cWL) provided by LEDs (**Figures S1D and S2C**) as control conditions for the rest of the study.

**Figure 1:**
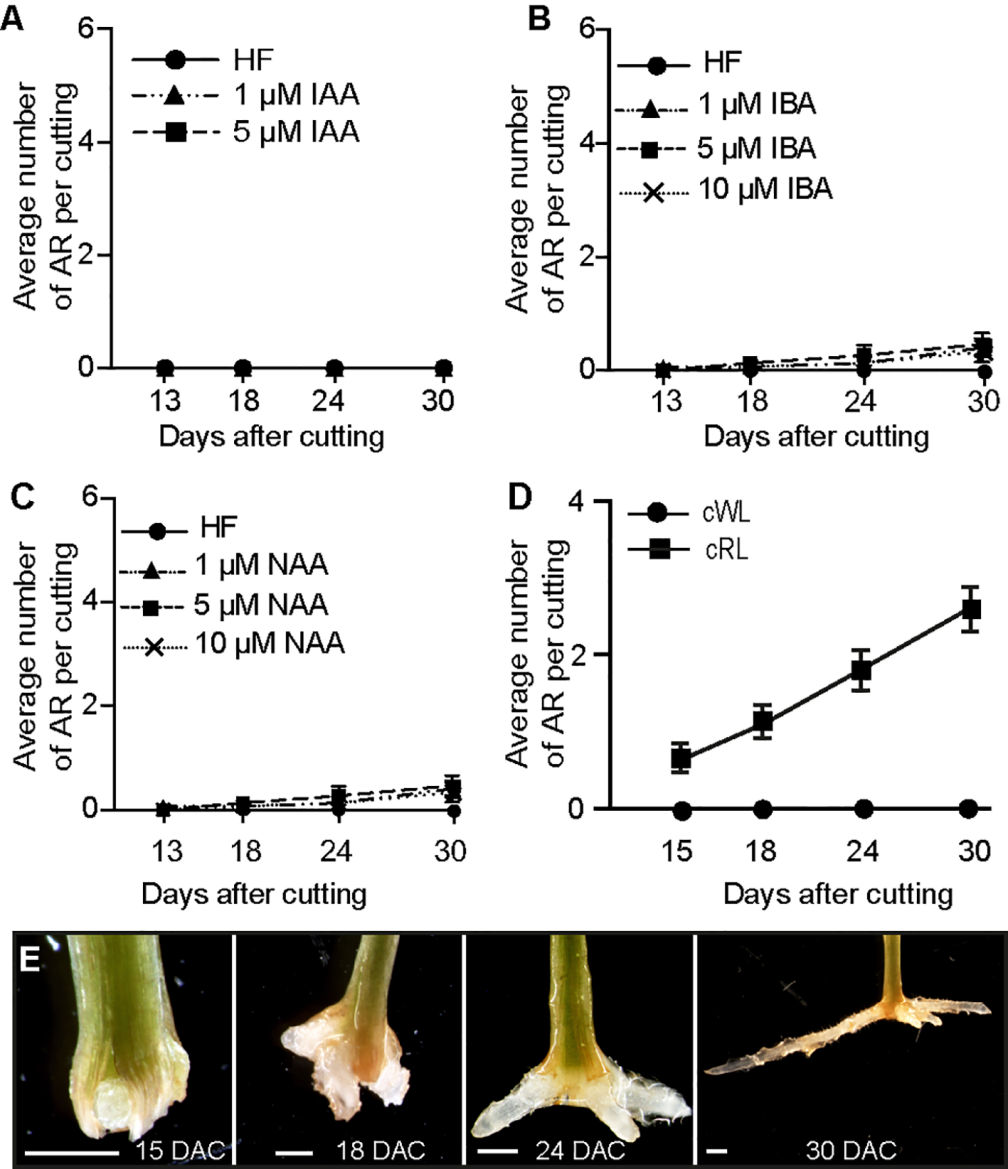
Red light promotes ARI in de-rooted Norway spruce hypocotyls. **(A**, **C)** Three-week-old Norway spruce seedlings were de-rooted and kept under cWL for 30 days in in hormone free (HF) distilled water, or in the presence of 1 or 5 μM IAA **(A)** or in the presence of 1, 5 or 10 μM IBA **(B)** or NAA **(C)**. **(A)** Exogenously applied IAA did not have any effect. **(B)** Three concentrations of IBA did not have any significant effect compared to HF conditions (Repeated measures ANOVA; p = 0.23). **(C)** Three concentrations of NAA did not have any significant effect compared to HF conditions (Repeated measures ANOVA; p = 0.11). **(D)** Three-week-old Norway spruce seedlings were de-rooted and kept for 30 days in hormone free (HF) distilled water under cWL or cRL. The hypocotyl cuttings were observed every day to count emerging AR primordia and emerged ARs as in **(E)**. Fifteen seedlings were analyzed for each condition. Adventitious root number increased significantly (t-test, df = 3, p < 0.0001) by cRL compared to cWL over time. **(E)** Different stages of AR development in de-rooted 3-week-old Norway spruce seedlings in distilled water, under cRL. DAC: days after cut; scale bar = 2 mm.

It is well known that exogenously applied auxin can stimulate adventitious rooting in several plant species (Lakehal and Bellini, 2019), therefore we tested whether different types of auxin could stimulate ARI under cWL. Three-week-old de-rooted seedlings were treated with 1 or 5 µM of Indole 3-Acetic Acid (IAA), 1, 5 or 10 µM 1-Naphthalene 3-Acetic Acid (1-NAA) or Indole 3-Butyric Acid (IBA), but none of the exogenously applied auxins could induce ARI under cWL conditions (**Figures 1A–C**). These results indicate that auxin is not sufficient to induce ARI in de-rooted Norway spruce seedlings kept under cWL.

### Constant Red Light (cRL) Promotes ARI in De-Rooted Norway Spruce Hypocotyls

Red light (RL) has long been used to promote adventitious rooting in recalcitrant species (reviewed in Christiaens et al., 2016), but the exact molecular mechanism underlying its effect is still largely elusive. Hence, we wondered whether RL could promote ARI on Norway spruce seedlings. To test this hypothesis, 3-week old de-rooted seedlings were kept in HF distilled water under either cWL (**Figures S1D and S2C**), or constant red light (cRL) (**Figures S1E and S2D**). Interestingly, AR developed only when seedlings were kept under cRL conditions (**Figures 1D, E**). In these conditions 100% of the cuttings developed at least 1 AR by 30 days after cutting (DAC) (**Figure 1D**). These data indicate that cRL promotes ARI even in the absence of exogenously applied auxin possibly through repressing the negative regulators of ARI such as JA or CKs.

### Constant Red Light Induces ARI by Reducing Endogenous Levels of Jasmonate and Cytokinins

**Figure 2:**
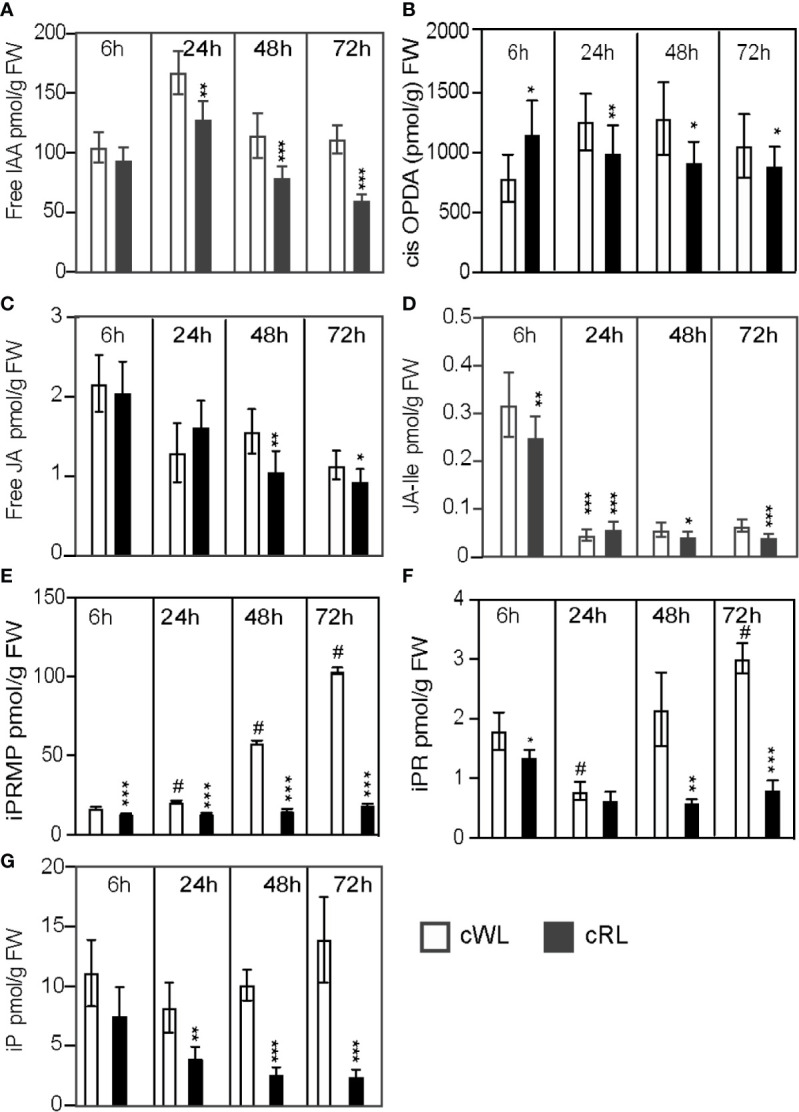
Light spectral quality controls the homeostasis of endogenous contents of different hormones, which positively or negatively affect ARI. **(A–G)** Three-week-old Norway spruce seedlings were grown under long day conditions as described in *Materials and Methods*. De-rooted seedlings were then kept in constant cWL or cRL for 6, 24, 48, and 72 h. For each time point in each condition 5 mm hypocotyls were taken and pooled for hormone quantification. Five independent biological replicates were collected for analysis of IAA, and JA contents and additional five independent biological replicates were collected for CKs quantification. **(A)** free IAA content; **(B)** free *cis*-12-oxo-phytodienoic acid (*cis*-OPDA) content; **(C)** free jasmonic acid (JA) content; **(D)** jasmonate-isoleucine (JA-Ile) content; **(E)** iP riboside 5′-monophosphate (iPRMP) content; **(F)** iP riboside content (iPR); **(G)** isopentyl-adenine (iP) content. Values are the means with standard deviation (SD) of 5 biological replicates. Asterisks indicate statistically significant differences under light conditions cRL versus cWL in t-test; *, **, and *** correspond to P-values of (0.05 > p > 0.01, 0.01 > p > 0.001, and p < 0.001 respectively; *n = 5*); Dash (#) ndicates statistically significant difference at 24 h, 48 h or 72 h versus 6 h in t-test; p < 0.001, n = 5; FW, fresh weight.

We have previously shown that ARI in intact etiolated Arabidopsis hypocotyls is under the control of IAA and JA signaling pathways (Gutierrez et al., 2012). More recently we showed that JA and CK cooperatively repress this process (Lakehal et al., 2020a). We therefore hypothesized that light might also modulate phytohormone signaling and/or homeostasis pathways to control ARI in Norway spruce hypocotyls. To test this hypothesis, we quantified the endogenous contents of different hormones known to either inhibit ARI, such as JA or CKs (Steffens et al., 2006; Ramírez-Carvajal et al., 2009; Gutierrez et al., 2012; McAdam et al., 2016; Mao et al., 2019) or promote ARI, such as IAA (Gutierrez et al., 2012). Quantifications were performed for hypocotyl samples taken at time 0 (T0), i.e. just before being transferred in HF distilled water, and at the base of hypocotyl cuttings maintained under different light qualities 6, 24, 48, and 72 h after cutting (**Figure 2**; **Supplemental Data Sets 1 and 2**).

Under cWL conditions, we observed a 40% increase in the free IAA level 6 h after cutting compared to the level at T0 **(** **Supplemental Data Set 1**), but thereafter the IAA content remained mostly constant over time, with the exception of a slight increase 24 h after cutting (**Figure 2A**; **Supplemental Data Set 1**). Based on these results, we concluded that the inability to initiate ARs under cWL could not be explained by a reduction in auxin content, and this is in agreement with the fact that exogenous application of IAA or other auxins could not stimulate ARI under these conditions (**Figures 1A–C**).

We have previously shown that JA and its bioactive form Jasmonoyol-Isoleucine (JA-Ile) inhibit ARI in Arabidopsis hypocotyls (Gutierrez et al., 2012; Lakehal et al., 2020a). Here, as expected in the case of wounding, JA and JA-Ile were induced 6 h after cutting in all light conditions (**Supplemental Data Set 1**). Under cWL conditions, we observed a 93% increase in both JA and JA-Ile 6 h after cutting and transfer to cWL conditions (**Supplemental Data Set 1**). The JA precursor *cis*-12-oxo-phytodienoic acid (*cis*-OPDA), JA and JA-Ile contents subsequently decreased over time (**Figures 2B–D**; **Supplemental Data Set 1**). Interestingly, although we observed a significant decrease (72%) in total CK content 6 h after cutting compared to the content at T0 (**Supplemental Data Set 2**), the total CK content then increased again and CKs accumulated over time at the base of the hypocotyls (**Figure 2**). This increase in CKs was mostly due to the accumulation of isopentyl-adenine-type (iP-type) cytokinins including the precursors iP riboside 5′-monophosphate (iPRMP) and iP ribosides (iPR) (**Figures 2E–G**; **Supplemental Data Set 2**).

Interestingly, in cRL conditions, although the free IAA content was significantly reduced compared to that in cWL 24 h after cutting and continued to decrease over time (**Figure 2A**), cutting developed ARs. This could be explained by a significant reduction in the amount of JA, Ja-Ile and CKs compared to cWL at all time points (**Figures 2C–G**). In contrast to what was observed in cWL, the amounts of CKs remained constant over time, except for iP which continued to decrease over time (**Figure 2G**). Although the interaction between light spectral quality and hormone homeostasis is complex, these results suggest that the positive effect of cRL on ARI cannot be explained by the modification of IAA homeostasis**;** rather it is a consequence of a decrease in negative regulators such as JA, JA-Ile, and iP.

### Exogenously Applied JA and CK Inhibit ARI Under cRL

To get physiological insights into the possible crosstalk between IAA, JA and CKs during cRL-induced AR development, we treated the de-rooted seedlings exogenously with either auxins, JA, or CK alone or in combination.

**Figure 3:**
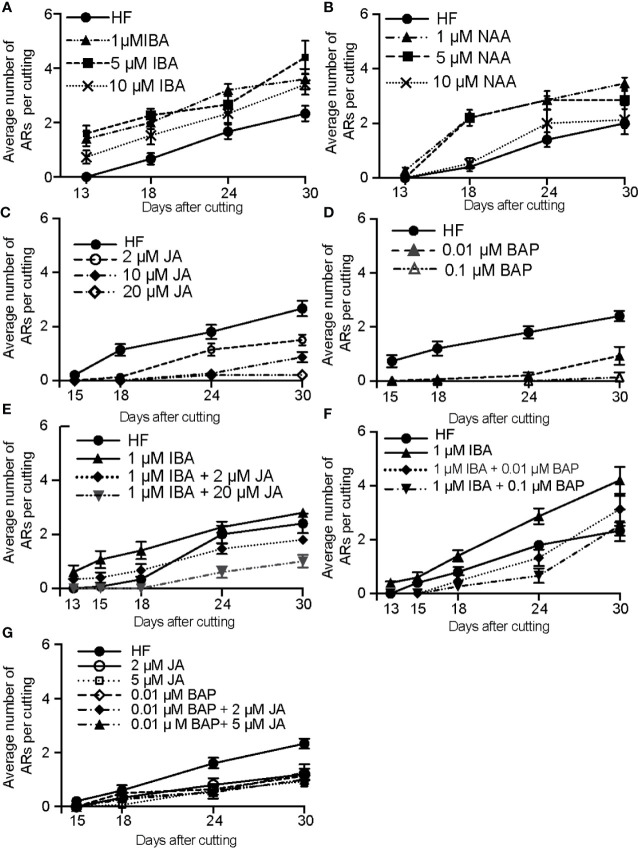
Exogenous application of auxin enhances RL-induced adventitious rooting, whereas exogenously applied CKs and JA repress this process. Three-week-old Norway spruce seedlings were de-rooted and kept under cRL for 30 days in hormone free (HF) distilled water, or in the presence of 1 or 5 μM IBA **(A)**; or in the presence of 1 or 5 µM NAA **(B)** or in the presence of jasmonate (JA) **(C)**; or in the presence of benzyl adenine (BAP) **(D)**; or in the presence of both JA and IBA **(E)**; or in the presence of both BAP and IBA **(F)**; or in the presence of both JA and BAP **(G)**. **(A)** All three concentrations of exogenously applied IBA significantly increased the average number of ARs over time (repeated measures ANOVA; P < 0.0001). **(B)** One or 5 µM of exogenously applied NAA significantly increased the average number of adventitious roots over time (repeated measures ANOVA; p < 0.0001) but no significant improvement compared to HF conditions was observed when 10 μM of NAA were applied (Repeated measures ANOVA; p = 0.48). **(C)** Exogenously applied JA had a significant negative effect on the average number of ARs over time (repeated measures ANOVA; p < 0.0001). This negative effect was concentration dependent (repeated measures ANOVA; p < 0.0001). **(D)** Exogenously applied BAP significantly reduced the number of ARs compared to HF conditions (repeated measures ANOVA; p < 0.0001). **(E)** When JA was applied together with IBA, it significantly repressed the positive effect of IBA. The combination 1 μM IBA + 2 μM JA significantly reduced the number of roots compared to those in HF conditions by 24 days after cut (DAC) and 30 DAC (t-test, degree of freedom df = 14; p = 0.02 and 0.001 respectively). The combination 1 μM IBA + 20 μM JA significantly reduced the average AR number compared to HF conditions (t-test, df = 14; p < 0.01). **(F)** When BAP was applied together with IBA, it repressed the positive effect of IBA. The combination 1 μM IBA + 0.01 μM BAP significantly reduced the average number of ARs compared to the 1 μM IBA treatment. (13, 15, 18, 24, and 30 DAC; t-test, df = 14; p < 0.009, 0.007, 0.003, 0.0001 and 0.02, respectively). The combination 1 μM IBA + 0.1 μM BAP significantly inhibited rooting induced by 1μM IBA at (13,15,18,24 and 30 DAC (t-test, df = 14; p < 0.008,0.007,0.0009, 0.000000009 and 0.0009 respectively); **(G)** Both JA and BA repressed AR compared to the HF control, but no significant additive or synergistic effect was observed when JA and BAP were applied together. The combination 0.01 μM BAP + 2 μM JA significantly inhibited rooting compared to the HF control at 24 and 30 DAC (t-test, df = 14; p < 0.0007 and 0.003 respectively), but the result was not significantly different from JA or BAP alone; The combination of 0.01 μM BAP + 5μM JA significantly inhibited rooting compared to the HF controls at 24 and 30 DAC (t-test, df = 14; p < 0.00004 and 0.0000003 respectively), but with no significant difference from JA or BAP alone. **(A**–**G)** Results are expressed as average number of ARs per cutting. Error bars indicate standard error (SE). For each treatment 15 hypocotyls were used, and the experiment was repeated twice.

First, we showed that the two types of exogenously applied auxin (IBA and NAA) enhanced AR formation under cRL conditions in a dose-dependent manner (**Figures 3A, B**). IBA, which is often used to induce rooting in recalcitrant species (reviewed in Geiss et al., 2018; Stevens et al., 2018), appeared to be the most efficient auxin (**Figure 3A**). When applied exogenously, both JA and 6-Benzylaminopurine (6-BAP) inhibited AR development in a dose-dependent manner (**Figures 3C, D**) and they both repressed the positive effect of exogenous IBA (**Figures 3E, F**). These results suggest that JA and CK act downstream of auxin signaling to repress ARI, as has been described for intact Arabidopsis hypocotyls (Gutierrez et al., 2012; Lakehal et al., 2020a). When JA and CK were combined, no additive or synergistic effect was observed (**Figure 3G**), suggesting that they act in the same pathway. These results are in agreement with our previous data that showed that CK signaling is induced by JA to repress AR formation in intact Arabidopsis hypocotyls (Lakehal et al., 2020a).

### JA Signaling Is Downregulated in De-Rooted Norway Spruce Hypocotyls Kept Under cRL Compared to cWL Conditions

The reduction in content of JA and JA-Ile under cRL compared to cWL (**Figure 2B**) prompted us to check whether the expression of genes involved in JA biosynthesis or JA signaling was affected in hypocotyls of seedlings kept under cRL compared to cWL conditions. We first searched the Norway spruce genome (Nystedt et al., 2013) for putative orthologs of the key Arabidopsis genes in JA signaling or biosynthesis using http://congenie.org/. We looked for putative orthologs of the JA-Ile receptor *CORONATINE INSENSITIVE 1 (AtCOI1)* (Xie et al., 1998)*, AtMYC2* transcription factors (Lorenzo et al., 2004), the *JASMONATE ZIM-DOMAIN3 (AtJAZ3) and AtJAZ10* transcriptional repressors (Chini et al., 2007; Thines et al., 2007; Yan et al., 2007) and *ALLENE OXYDE CYCLASE (AtAOC*), which encodes a key enzyme in the JA biosynthesis pathway (Stenzel et al., 2012). We found that the Norway spruce genome contains eleven putative *PaCOI1-like* genes (**Figure S3A**), five putative *PaMYC2-like* genes (**Figure S3B**), one and three putative orthologs of *PaJAZ3-like* and *PaJAZ10-like* respectively (**Figure S3C**) and two putative *PaAOC-like genes* (**Figure S3D**). The full-length coding sequences are reported in **Figure S4**. When more than one putative ortholog was identified we chose that most closely related to the Arabidopsis one for further experiments.

**Figure 4:**
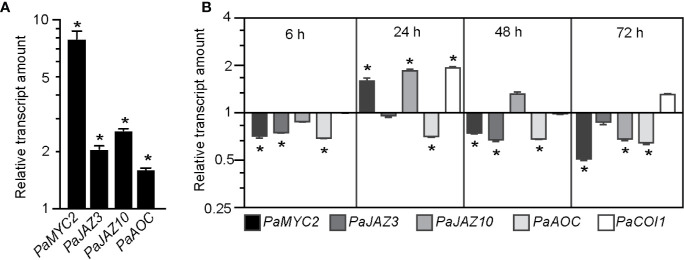
JA signaling is downregulated in hypocotyl cuttings kept under constant red light (cRL) compared to hypocotyl cuttings kept under constant white light (cWL). **(A)** Relative transcript amount of putative JA-responsive genes *PaMYC2-like*, *PaJAZ3-like*, *PaJAZ10-like* and *PaAOC-like* in 3-week-old Norway spruce seedling hypocotyls treated for 3 h with 50 μM JA or with a mock treatment. Values are relative (on a log10 scale) to the values for mock treated seedlings which were arbitrarily set to 1. Error bars indicate ±SE obtained from two independent biological replicates, with three technical replicates of each. A *t*-test was carried out, and values indicated by asterisks differed significantly from the mock values (p < 0.001, n ≥ 80). **(B)** Relative transcript amounts of putative JA-responsive genes *PaMYC2-like*, *PaJAZ3-like*, *PaJAZ10-like* and *PaAOC-like*, and the putative JA receptor *PaCOI1-like*, at the base of 3-week-old de-rooted hypocotyls kept under cRL or cWL for 6, 24, 48 and 72 h. The values are relative (on a log2 scale) to the values obtained from seedlings kept under cWL, which were arbitrarily set to 1. Error bars indicate ±SE obtained from two independent biological replicates, with three technical replicates of each. A *t*-test was carried out, and asterisks indicate values that differed significantly from the control values (p < 0.001, n ≥ 80).

We first confirmed that expression of *PaMYC2-like* (MA_15962g0010), *PaJAZ3-like* (MA_6326g0010), *PaJAZ10-like* (MA_10229741g0010) and *PaAOC-like* (MA_56386g0010) was induced by exogenously applied JA (**Figure 4A**). We next checked the expression of these genes, together with *PaCOI1-like*, at the base of hypocotyl cuttings kept under cWL or cRL for 6 h, 24 h, 48 h or 72 h after cutting (**Figure 4B**). The relative amounts of *PaMYC2-like*, *PaJAZ3-like*, and *PaAOC-like* transcripts were slightly reduced in cRL compared to those observed in cWL (**Figure 4B**). These results are in agreement with the reduced content of JA-Ile, the active form of JA, in cRL compared to cWL (23% less in cRL compared to cWL) (**Figure 2F** and **Supplemental Data Set 2**). Twenty-four hours after cutting, *PaMYC2-like*, *PaJAZ10-like*, and *PaCOI1-like* were slightly upregulated in cRL compared to cWL (**Figure 4B**), results which also coincided with a slightly higher concentration of JA-Ile (20% more in cRL compare to cWL) (**Figure 2F**). Later on, by 48h and 72h, the JA responsive genes were downregulated (**Figure 4**), and this coincided with a greater decrease in JA and JA-Ile contents in cRL compared to cWL (**Figure 2F**; **Supplemental Data Set 2**). In conclusion, when de-rooted hypocotyls were kept in cRL conditions, the endogenous contents of JA and JA-Ile decreased faster than in cWL, resulting in downregulation of JA signaling in cRL compared to cWL conditions. According to our previous results (Gutierrez et al., 2012; Lakehal et al., 2020a), this most likely contributes to an improvement in adventitious root development.

### Exogenously Applied JA Inhibits ARI in Norway Spruce Hypocotyl in cRL

**Figure 5:**
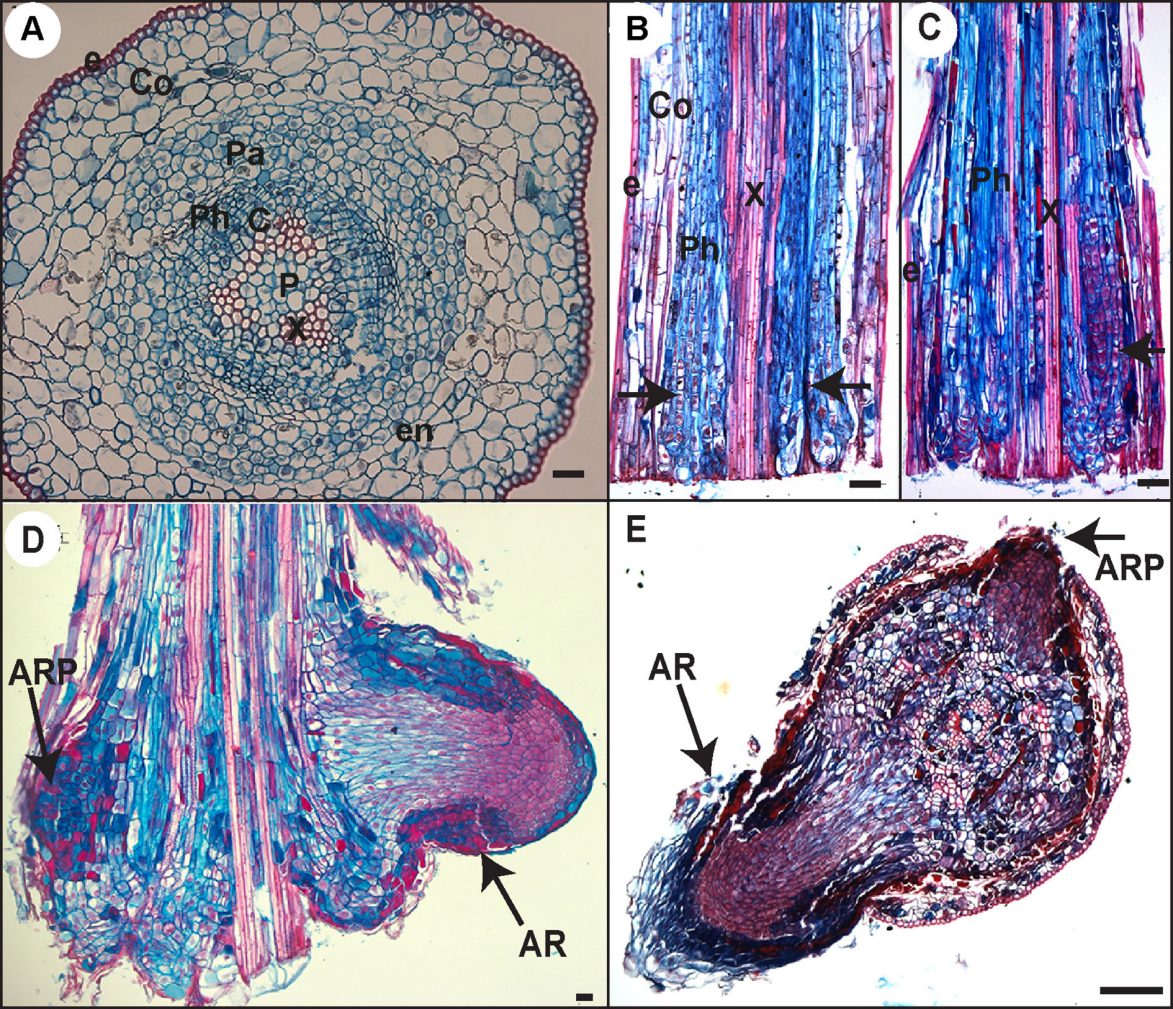
Histological analysis of AR development in Norway spruce hypocotyl cuttings under cRL. **(A)** Cross section through the base of a 21-day-old hypocotyl cutting just before transfer to rooting conditions. **(B)** Longitudinal section through the base of a hypocotyl cutting kept in hormone free (HF) conditions for 5 days. Arrowheads indicate localized cell divisions, probably at the points of origin of future ARs. **(C)** Longitudinal section through the base of a hypocotyl cutting kept in the presence of 1 μM IBA for 10 days. Arrowheads indicate the presence of clusters of dividing cells. **(D)** Longitudinal section through the base of a hypocotyl cutting kept in the presence of 1 μM IBA for 13 days. Arrows indicate an adventitious root primordium (ARP) and an emerged adventitious root (AR). **(E)** Cross section through the base of a hypocotyl cutting kept in HF conditions for 15 days. Arrows indicate an ARP and an emerged AR. e, epidermis; Co, cortex; Pa, parenchyma; Ph, phloem; en, endodermis; c, cambium region; P, pith; x, xylem. In all panels scale bars = 20 μm.

**Figure 6:**
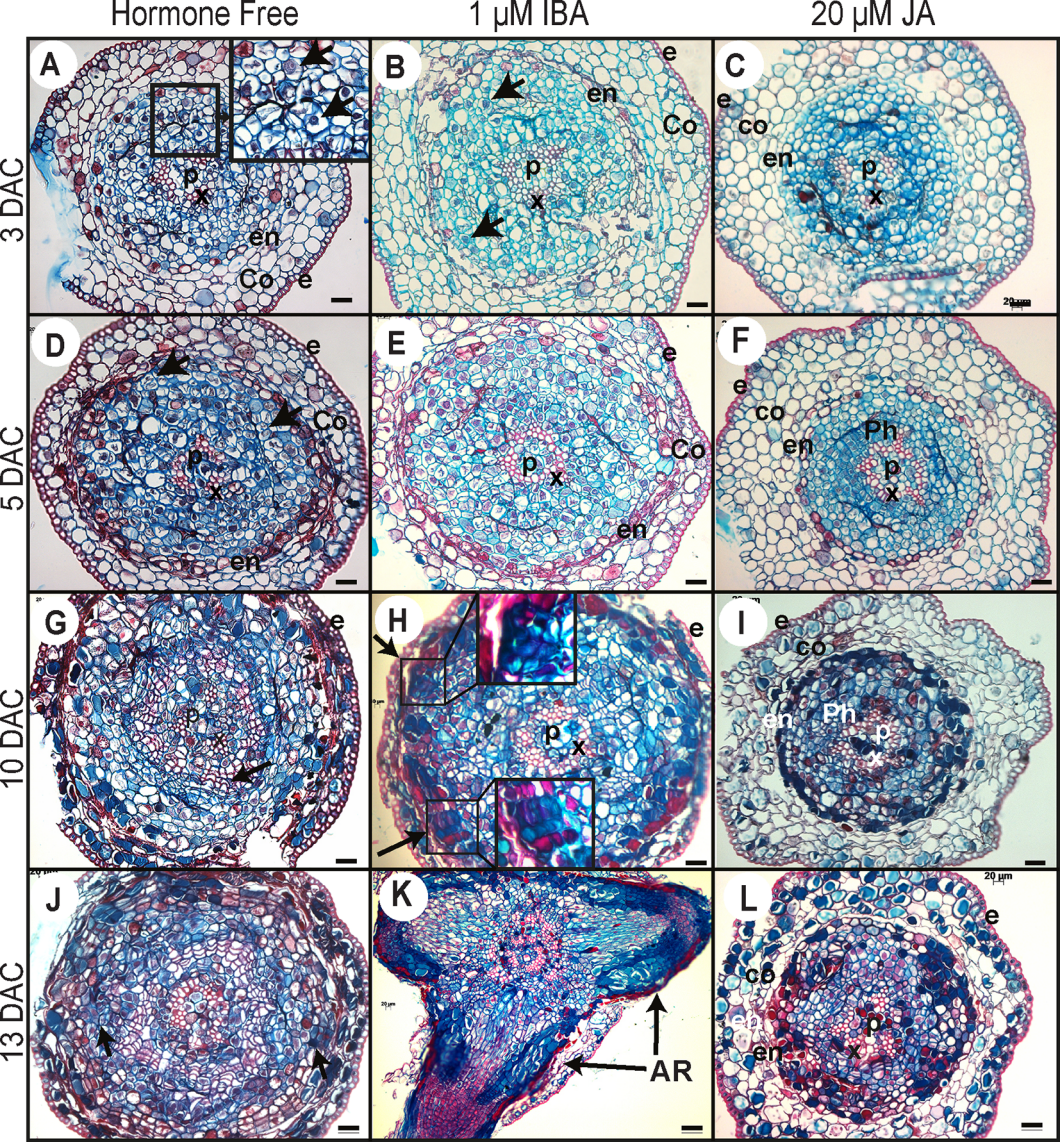
Histological analysis of ARI in Norway spruce hypocotyl cuttings under cRL in the presence of IBA, JA or HF. **(A, D, G, J)** Cross sections were prepared from the base of de-rooted Norway spruce hypocotyl cuttings, kept under cRL in hormone free (HF) distilled water for 3, 5, 10, or 13 days. In **(A)**, the arrows show early cell activation, *i.e*. small cells with dense cytoplasm and large nuclei. In **(D)**, arrows indicate anticlinal and periclinal division. In **(G)** arrows show the presence of tracheal elements. In **(J)**, arrows indicate clusters of dividing cells developing at the periphery of the tracheal elements. **(B, E, H, K)** Cross sections were taken from the base of de-rooted Norway spruce hypocotyl cuttings, kept under cRL in the presence of 1 μM IBA for 3, 5, 10, or 13 days. In **(B)** arrows indicate cell divisions; In **(H)** arrows indicate the presence of clusters of dividing cells for which close-up views are shown. In **(K)** arrows indicate emerged adventitious roots (ARs). **(C, F, I, L)** Cross sections were prepared from the base of de-rooted Norway spruce hypocotyl cuttings, kept under cRL in the presence of 20 μM JA for 3, 5, 10, or 13 days. e, epidermis; Co, cortex; Ph, phloem; en, endodermis; c, cambium region; P, pith; x, xylem. In all panels scale bars = 20 μm.

Our data indicated that JA signaling was rapidly downregulated (within 6 h) in the hypocotyls of cuttings kept under cRL compared to cWL, suggesting that JA signaling repressed an early event in AR formation. To assess the repressive effect of JA at the cellular level, we analyzed and compared the anatomy of hypocotyls kept under cRL, in hormone free distilled water or in the presence of 1 µM IBA or 20 μM JA, at different time points after cutting (**Figures 5** and **6**). On day 0 (i.e. immediately after cutting the hypocotyl of 3-week-old Norway spruce seedlings), the hypocotyl had a primary structure consisting of a single layer of epidermis, and six to seven cortex cell layers composed of isodiametric parenchyma cells (**Figure 5A**). We could guess at the presence of the endodermis layer although it was not always morphologically distinctive (**Figure 5A**). The central cylinder consisted of seven to eight layers of parenchyma cells which were smaller compared to the cortex parenchyma cells. The vascular system was composed of a continuous procambium and differentiated elements of the protophloem and protoxylem. The pith region showed small isodiametric parenchyma cells (**Figure 5A**).

Three days after cutting, in HF distilled water and in the presence of 1 µM IBA, a few cells in the cambial zone and in the adjacent phloem cells developed dense cytoplasm and large nuclei indicating the initiation of the first cell divisions (**Figures 6A, B**). In contrast, no obvious histological modifications were observed in the presence of 20 μM JA, and the histological organization remained unchanged compared to day 0 (**Figure 6C**).

By 5 days after cutting, periclinal and anticlinal divisions were clearly observed in the cambial zone and in the outermost layers of the phloem region (**Figure 6D**). In longitudinal section these cells were seen to be organized into vertical files external, but adjacent, to the vascular cylinder (**Figure 5B**). In the presence of 1 μM IBA it was clearly apparent that the number of cell divisions was higher than under HF conditions (**Figure 6E**), whereas no divisions were observed in the presence of 20 μM JA (**Figure 6F**).

Ten days after cutting, in HF conditions, cambial derivatives divided and formed radial rows of tracheal elements around the xylem, but no AR clusters of dividing cells were observed (**Figure 6G**), while in the presence of 1 μM IBA several AR clusters of dividing cells were visible (**Figures 5C** and **6H**); no cell divisions were observed in the presence of 20 μM JA (**Figure 6I**). It was only by 13 days after cutting that AR clusters of dividing cells began to form at the periphery of the trachea elements in HF conditions (**Figure 6J**), while in the presence of IBA, AR had already emerged (**Figures 5D** and **6K**); still no cell division activity was observed in the presence of JA (**Figure 6L**). In HF, the first emergence of ARs was observed only 15 days after cutting (**Figure 5E**). These data clearly indicate that exogenously applied JA repressed the very early stage of ARI, whereas auxin not only promoted but also speeded up the process.

## Discussion

When optimizing conditions for the development of roots from cuttings during vegetative propagation, it has long been considered that light should be taken into account as an important factor. Since the early eighties, studies related to the effect of light on AR formation in woody plant cuttings have been pursued (Druart et al., 1982; Hammerschlag et al., 1987; Klopotek et al., 2010). Several studies have addressed the effect of light quality and/or intensity on the rooting of cuttings (Jarvis and Shaheed, 1987; Baraldi et al., 1988; Fuernkranz et al., 1990; Fett-Neto et al., 2001; Iacona and Muleo, 2010; Daud et al., 2013), but the exact molecular and genetic mechanisms underlying the role of light as well as the downstream targets of light signals during ARI remain poorly understood. In this study, we showed that under cWL conditions de-rooted Norway spruce hypocotyls were unable to produce AR and that this was unlikely to be due solely to an increase in endogenous CK levels, as suggested by (Strömquist and Eliasson, 1979; Bollmark and Eliasson, 1990) since exogenously applied auxin was unable to stimulate AR development.

In contrast to cWL, cRL promoted ARI in de-rooted Norway spruce hypocotyls even in absence of exogenously applied auxin. Although a positive effect of RL AR formation has been observed for propagation of in vitro shoots of grapevines (*Vitis spp.*) (Poudel et al., 2008), *Ficus benjamina* (Gabryszewska and Rudnicki, 1997) and *Jatropha curcas* L. (Daud et al., 2013) or *Morinda citrifolia* *in vitro* leaf explants (Baque et al., 2010) but how and through which molecular and genetic networks it acts is still unknown. RL has been shown to promote auxin polar transport in several species (Liu et al., 2011; reviewed in Sassi et al., 2013) and more precisely to promote lateral root development in tobacco (Nicotiana tabacum) seedlings by shifting auxin distribution (Meng et al., 2015). In the case of Norway spruce de-rooted hypocotyls, our data suggest that the positive effect of RL is unlikely due to an increase of auxin content since the de-rooted seedlings kept under cRL accumulated less IAA compared to those kept under cWL during the early stages of ARI. The cRL-mediated ARI is rather due to a faster reduction in JA and JA-Ile contents compared to cWL. cRL also repressed the accumulation of CKs which was observed under cWL. Reduction in the amounts of these negative regulators to below a certain threshold led to a de-repression of ARI. The reduced amount of JA and JA-Ile in the base of cuttings could be partially explained by downregulation of *de novo* JA biosynthesis, since we observed a reduced amount of the JA precursor *cis*-12-oxo-phytodienoic acid (*cis*-OPDA) under cRL compared to cWL conditions 24, 48 and 72 h after cutting (**Figure 2B**). This result is supported by the downregulation of expression of *PaAOC-like*, a key gene in the JA biosynthesis pathway. The link between light and the JA signaling pathway has been proposed in several contexts, but how it affects JA biosynthesis is not yet known. Interestingly, several reports have revealed mechanistic insights into the roles of RL or red/far-red light ratio in the control of plant development or in adjusting growth-defense trade-offs (Kazan and Manners, 2011). For example, (Campos et al., 2016) found that mutating the RL-receptor *Phytochrome B* (*PhyB*) suppressed the stunted rosette phenotype of the *JazQ* mutant, which harbors five null mutations in JAZ family members. These data suggest that *PhyB*-mediated light signaling interacts with JA signaling to control growth in Arabidopsis. Under our conditions, we found that the expression levels of *PaMYC2-like*, a master transcriptional regulator of JA signaling, were slightly but significantly downregulated under cRL compared to cWL. This result could be explained either by a direct effect of RL on transcriptional regulation or by the reduced amount of the bioactive form JA-Ile present under these conditions. The latter assumption is supported by the fact that exogenous application of JA strongly induced the expression of *PaMYC2-like*, an observation consistent with what has been reported in Arabidopsis. The reduced amounts of JA and JA-Ile and the downregulation of the expression of *MYC2-like* under cRL conditions led to a reduced JA response, as indicated by downregulation of the steady state level of expression of the well-known JA responsive genes (*PaJAZ10-like* and *PaJAZ3-like*).

Although the negative role of JA on ARI has been demonstrated in the model plant Arabidopsis, its exact role in other species previously remained unclear (reviewed in (Lakehal et al., 2020b). Our results showed that JA is a negative regulator of ARI in de-rooted Norway spruce hypocotyl and that it acts during the very early stages of initiation, as indicated by anatomical characterization. We showed that under cRL, certain cells, most likely cambial cells, have the competence to form ARs in a similar way to that described for other conifers such as *Pinus taeda*, *P. contorta* and *P. radiata* (Diaz-Sala et al., 1996; Lindroth et al., 2001a; Lindroth et al., 2001b; Ricci et al., 2008). When we exposed the de-rooted hypocotyls to exogenous auxin, these cells exhibited rapid cell division and re-orientation of division planes to organize the AR meristem**;** in contrast when exogenous JA was added, no cell divisions were observed. In addition, we showed that JA repressed the positive effect of auxin when the compounds were applied together, supporting the conclusion that, as in Arabidopsis, JA acts downstream of auxin in the control of ARI in de-rooted Norway spruce hypocotyls. Moreover, we showed that, like JA, CK inhibits ARI and counteracts the action of auxin, but when combined with JA no additive or synergistic effect was observed, which is in agreement with our recent results showing that cytokinins act downstream of JA to repress ARI in Arabidopsis hypocotyls (Lakehal et al., 2020a).

Our data also showed that total CKs accumulated at the base of cuttings when the de-rooted seedlings were exposed to cWL, whereas their amount remained constant over time under cRL conditions. Under cWL, the increasing amounts of the iP-type CKs could be due to *de novo* biosynthesis since we also observed an increased level of the precursors iPR and iPRMP over time. (Bollmark and Eliasson, 1990) showed that de-rooted Norway spruce hypocotyls kept under high irradiance WL (270 µE.m^−2^ s^−1^) could not develop ARs, probably because of the accumulation of CKs. Nevertheless, the effect of WL may be more complex, since under our conditions, although the WL irradiance was much lower (75 µE m^−2^ s^−1^), CKs still accumulated. In our conditions, as a response to wounding, JA and JA-Ile accumulated at the base of hypocotyls 6 h after cutting, and their content started decreasing thereafter, whereas iP-type CKs began to accumulate 48 h after cutting. Recently we showed that MYC2-mediated JA signaling promotes the accumulation of CKs through control of expression of the *APETALA2/ETHYLENE RESPONSE FACTOR 115* transcription factor in Arabidopsis hypocotyls (Lakehal et al., 2020a). We therefore propose that in Norway spruce hypocotyls, wound induced accumulation of JA and JA-Ile triggers *de novo* cytokinin biosynthesis which results in inhibition of rooting. Interestingly, under constant cRL, the more rapid reduction in JA and JA-Ile contents and subsequent downregulation of MYC2-mediated JA signaling may prevent the induction of *de novo* CK biosynthesis and this would allow ARI, although we also cannot rule out a direct effect of cRL on the repression of CK accumulation.

In conclusion, we propose that de-rooted Norway spruce seedlings do not develop AR under WL, because they accumulate CKs which are repressors of ARI. In contrast, we showed that red light has a positive effect on ARI, most likely because it represses the JA signaling pathway and the accumulation of CKs. Nevertheless, further research is needed to unravel the exact signaling pathways mediating the effect of RL in promoting ARI in Norway spruce. The rapid development of sequencing methods and genome editing techniques will open up new opportunities to investigate the genetic and epigenetic mechanisms underlying ARI. The generation of an Epigenetic Plant Cell Atlas from Norway spruce hypocotyl could be an interesting future step for studying chromatin remodeling and other epigenetic regulatory pathways during ARI (Rhee et al., 2019).

## Data Availability Statement

Publicly available datasets were analyzed in this study. This data can be found here: http://congenie.org/.

## Author Contributions 

SA, AL, and CB conceived and designed the experiments. SA, AR, FB, ON, and AL performed the experiments. SA wrote the first draft of the manuscript. SA, AL, and CB reviewed and edited the manuscript. All authors contributed to the article and approved the submitted version. AL and CB supervised the work. SA, ON, and CB acquired funding.

## Funding

This research was supported by the Ministry of Higher Education and Scientific Research in Iraq (Grant n°30488) (to SA), by grants from the Swedish research councils FORMAS, VR, VINNOVA, Kempestiftelserna, and the Carl Tryggers Stiftelse (to CB), and by grants from the Ministry of Education, Youth and Sports of the Czech Republic (European Regional Development Fund-Project “Plants as a tool for sustainable global development” No. CZ.02.1.01/0.0/0.0/16_019/0000827), and the Czech Science Foundation (Project No. 19-00973S) (to ON).

## Conflict of Interest

The authors declare that the research was conducted in the absence of any commercial or financial relationships that could be construed as a potential conflict of interest.
