# Supplementary material for: Red Light Controls Adventitious Root Regeneration by Modulating Hormone Homeostasis in Picea abies Seedlings
Source: Front Plant Sci. 2020 Sep 9;11:586140. doi: 10.3389/fpls.2020.586140 (PMC7509059; doi:10.3389/fpls.2020.586140)
Supplement: Supplementary file 1 [file DataSheet_1.pdf]

Supporting Information to

Red light controls adventitious root regeneration by modulating hormone homeostasis in *Picea abies* seedlings

Sanaria Alallaq[1,2], Alok Ranjan[1,#] , Federica Brunoni[1,§] , Ondřej Novák[3,4], Abdellah Lakehal[1,*] , Catherine Bellini [1,5,*]

[1] Umeå Plant Science Centre, Department of Plant Physiology, Umeå University, SE-90736 Umeå, Sweden

[2] Department of Biology, College of Science for Women, Baghdad University,10071, Baghdad, Iraq

[3] Umeå Plant Science Centre, Department of Forest Genetics and Physiology, Swedish Agriculture University, SE-90183 Umea, Sweden

[4] Laboratory of Growth Regulators, Faculty of Science, Palacký University and Institute of Experimental Botany, The Czech Academy of Sciences, 78371 Olomouc, Czech Republic

[5] Institut Jean-Pierre Bourgin, INRAE, AgroParisTech, Université Paris-Saclay, FR-78000 Versailles, France

[#]**Present address:** Unité de recherche en Physiologie et Génétique moléculaire des Plantes. Faculté des Sciences Campus de la Plaine, 1050 Bruxelles, Belgium

[§] **Present address:** Laboratory of Growth Regulators, Faculty of Science, Palacký University and Institute of Experimental Botany, The Czech Academy of Sciences, 78371 Olomouc, Czech Republic

***Corresponding authors:**

Pr Catherine Bellini email: catherine.bellini@umu.se

Dr Abdellah Lakehal email: abdellah.lakehal@umu.se

The following Supporting Information is available in this PDF:

Figure S1: Method for *de novo* root organogenesis under different light conditions

Figure S2: Spectral emission curves for the various light sources used in this study

Figure S3: Phylogenetic trees

Figure S4: Coding sequences used for the phylogenetic analysis

Table S1 Primers used for qPCR experiments in this study

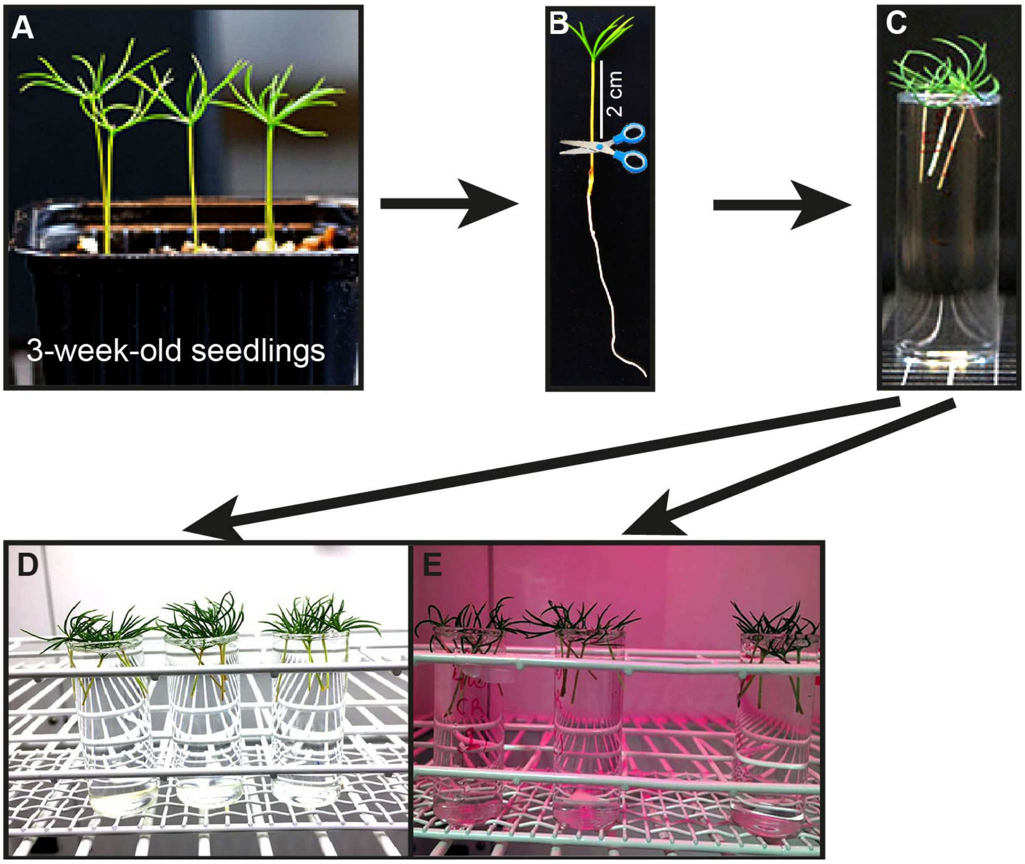

Figure. S1 Method for *de novo* root organogenesis under different light conditions

(A) Three-week-old Norway spruce seedlings grown in vermiculite in a growth chamber under long day conditions

(B) Three-week-old Norway spruce seedling before cutting.

(C) Hypocotyl cuttings transferred to 24 ml vials filled with distilled water and placed in monochromatic cabinets equipped with white (D) or red (E) LEDs

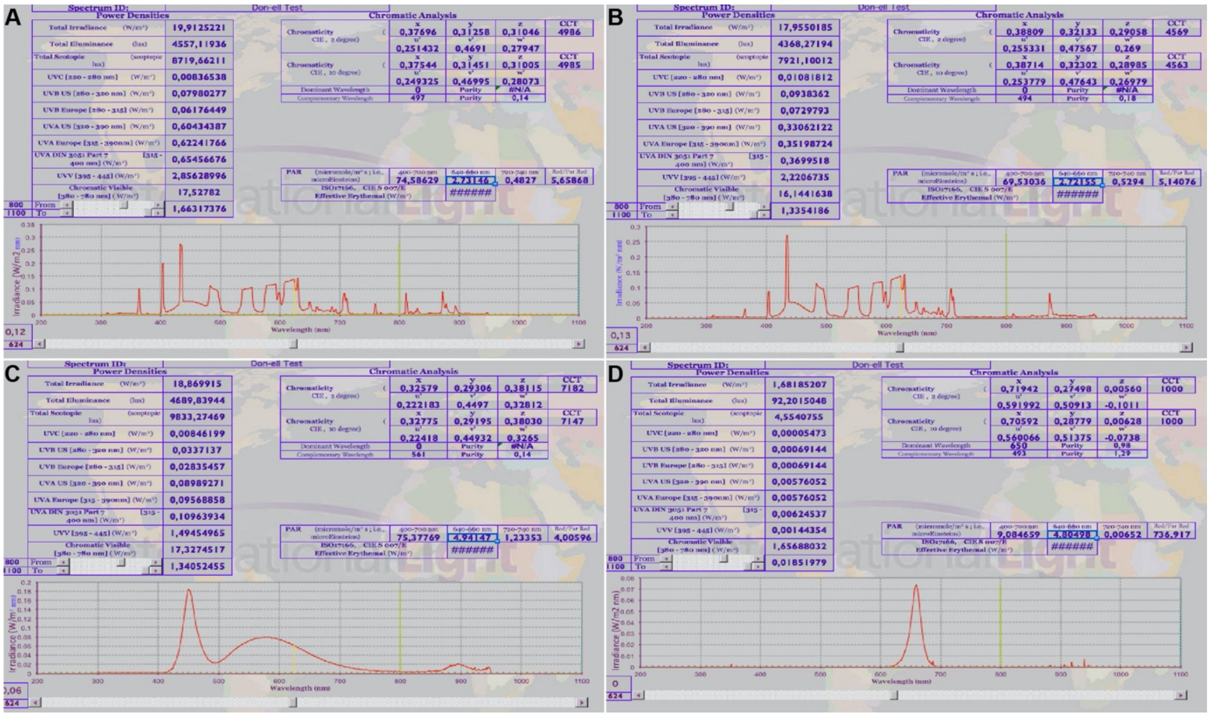

Figure S2: Spectral emission curves for the various light sources used in this study.

(A) White light TL-D 36W/840 Philips master, long days

(B) Cool white light F17T8/TL741, 17 W Philips, long days

(C) Continuous white light LED (cWL)

(D) Continuous red light LED (cRL).

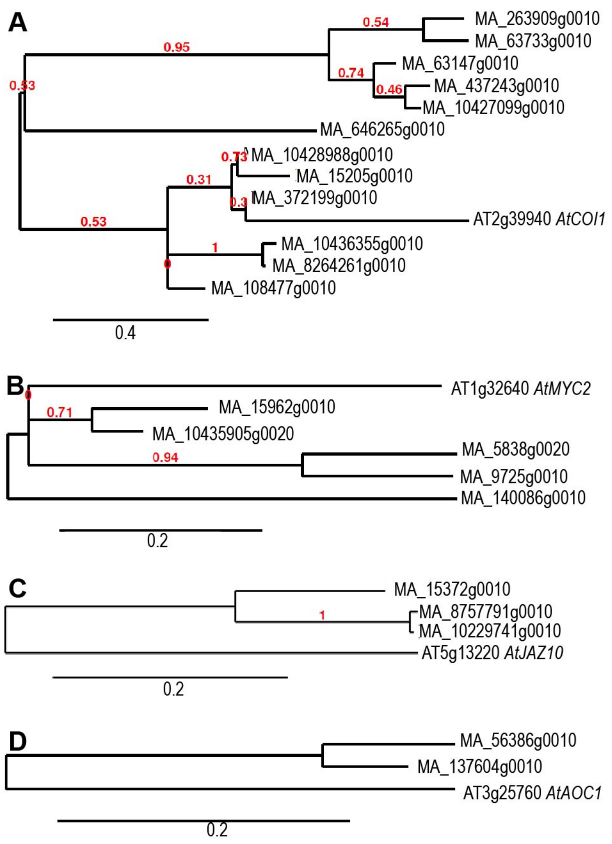

Figure S3 Phylogenetic trees

Neighbor-Joining phylogenetic trees (1000 bootstrap replicates), based on alignment of coding sequences of (A) *COI1* (B) *MYC2* (C) *JAZ10* and (D) *ACO1* (E) from *Picea abies* (MA) and *A. thaliana* (AT). The gene orthologs were named according to the http://congenie.org/ database. The sequences are provided in Figure S4.

Figure S4 Coding sequences used for the phylogenetic analysis

Arabidopsis thaliana and *Picea abies* coding sequences (CDS) were obtained from the TAIR (https://www.arabidopsis.org) and congenie (http://congenie.org/) databases respectively. To find putative Arabidopsis orthologs in *Picea abies*, a BLAST alignment of the coding sequences of selected genes was generated using "Genome Tools" at http://congenie.org/ with default settings. Subsequently, http://www.phylogeny.fr was used to construct phylogenetic trees based on the neighbor-joining method (with 1000 bootstrap replicates). Gene orthologs were named according to the http://congenie.org database.

> COI1_ AT2G39940

ATGGAGGATCCTGATATCAAGAGGTGTAAATTGAGCTGCGTCGCGACGGTTGATGATGTCAT
CGAGCAAGTCATGACCTATATAACTGACCCGAAAGATCGCGATTCGGCTTCTTTGGTGTGTC
GGAGATGGTTCAAGATTGATTCCGAGACGAGAGAGCATGTGACTATGGCGCTTTGCTACACT
GCGACGCCTGATCGTCTTAGCCGTCGATTCCCGAACTTGAGGTCGCTCAAGCTTAAAGGCAA
GCCTAGAGCAGCTATGTTTAATCTGATCCCTGAGAACTGGGGAGGTTATGTTACTCCTTGGG
TTACTGAGATTTCTAACAACCTTAGGCAGCTCAAATCGGTGCACTTCCGACGGATGATTGTC
AGTGACTTAGATCTAGATCGTTTAGCTAAAGCTAGAGCAGATGATCTTGAGACTTTGAAGCT
AGACAAGTGTTCTGGTTTTACTACTGATGGACTTTTGAGCATCGTTACACACTGCAGGAAAA
TAAAAACTTTGTTAATGGAAGAGAGTTCTTTTAGTGAAAAGGATGGTAAGTGGCTTCATGAG
CTTGCTCAGCACAACACATCTCTTGAGGTTTTAAACTTCTACATGACGGAGTTTGCCAAAAT
CAGTCCCAAAGACTTGGAAACCATAGCTAGAAATTGCCGCTCTCTGGTATCTGTGAAGGTCG
GTGACTTTGAGATTTTGGAACTAGTTGGGTTCTTTAAGGCTGCAGCTAATCTTGAAGAATTT
TGTGGTGGCTCCTTGAATGAGGATATTGGAATGCCTGAGAAGTACATGAATCTGGTTTTTCC
CCGAAAACTATGTCGGCTTGGTCTCTCTTACATGGGACCTAATGAAATGCCAATACTATTTC
CATTCGCGGCCCAAATCCGAAAGCTGGATTTGCTTTATGCATTGCTAGAAACTGAAGACCAT
TGTACGCTTATCCAAAAGTGTCCTAATTTGGAAGTTCTCGAGACAAGGAATGTAATCGGAGA
TAGGGGTCTAGAGGTCCTTGCACAGTACTGTAAGCAGTTGAAGCGGCTGAGGATTGAACGCG
GTGCAGATGAACAAGGAATGGAGGACGAAGAAGGCTTAGTCTCACAAAGAGGATTAATCGCT
TTGGCTCAGGGCTGCCAGGAGCTAGAATACATGGCGGTGTATGTCTCAGATATAACTAACGA
ATCTCTTGAAAGCATAGGCACATATCTGAAAAACCTCTGTGACTTCCGCCTTGTCTTACTCG
ACCGGGAAGAAAGGATTACAGATCTGCCACTGGACAACGGAGTCCGATCTCTTTTGATTGGA
TGCAAGAAACTCAGACGATTTGCATTCTATCTGAGACAAGGCGGCTTAACCGACTTGGGCTT
AAGCTACATCGGACAGTACAGTCCAAACGTGAGATGGATGCTGCTGGGTTACGTAGGTGAAT
CAGATGAAGGTTTAATGGAATTCTCAAGAGGCTGTCCAAATCTACAGAAGCTAGAGATGAGA
GGTTGTTGCTTCAGTGAGCGAGCAATCGCTGCAGCGGTTACAAAATTGCCTTCACTGAGATA
CTTGTGGGTACAAGGTTACAGAGCATCGATGACGGGACAAGATCTAATGCAGATGGCTAGAC
CGTACTGGAACATCGAGCTGATTCCATCAAGAAGAGTCCCGGAAGTGAATCAACAAGGAGAG
ATAAGAGAGATGGAGCATCCGGCTCATATATTGGCTTACTACTCTCTGGCTGGCCAGAGAAC
AGATTGTCCAACAACTGTTAGAGTCCTGAAGGAGCCAATATGA

> COI1-like_MA_10427099g0010
ATGCAGAGGCCGTGGAGGACGTCTGCGTGCGATGCGATTCCGGACGAGGTTTTGGAGTACGT
GATGGGGAACCTGGAAGACCCGCGGGATCGCAGCGCGGTTTCCCTGGTCTGCAAGAGCGCAC
GGTTCCCCAAGCTTGAGTCGCTGAAATTGAAGGGAAAGCCCAGGGCTGCCATGTTTAATTTG
ATTCCCCAGGATTGGGGCGGATACGCGAAGCCGTGGATTAACGAGATTTCCCAGAAATTTCT
CTGCTTAAAGGCTCTTCATTTGCGTAGAATGATCGTTACAAATGAGGATCTCGGGGTTCTGG
CTTGTGCCCGCGGCCACATTCTGCAGGTTCTTAAGCTGGAGAAGTGCTCGGGGTTTTCGACT
CTCGGGCTTCTCGAAGTCGCACGGTCCTGCAGGTAA

> COI1-like_MA_10428988g0010

ATGGAAATCCAGCGAGCGATGACGCTGGGCAACGAAATGGCAGAAGAAGCCCTGGAGTGCGT
GATGGATTACGTCAATGATCCCCGGGATCGAAGCGTGGTCTCTCAGGTGTGCAAGCAGTGGT
ACATGATCGATGCACTGACCCGAAAGCACGTGACCGTGGCCTTCTGCTACACAATCAAGCCC
GCCGATCTCACTCGGCGCTTCAGGCGGTTGGAGTCGTTGAAGCTGAAGGGGATGCCGAGGGC
CGCTATGTTCAACCTCATCCCAGAGGACTGGGGCGCCTACGCCTCCCCTTGGATTGAAGAGA
TCTCCTCTTCCTGCCTCTGCCTCAAGTCCCTCCACTTGCGGAGAATCATCGTCAAGGACGCC
GACCTCGCCACGCTCGTCGGAAGCCACGGTCAGATGCTGCAGGCCCTCAAGCTCGAGAAGTG
CTGCGGGTTCTCCACTCTCGGACTTCTGGAGATCTCTCGCTCTTGCAGATCTCTTAAGGTCT
TGTTTTTAGAGGAGAGTGAAATAAAAGATGAAGGTGGGGAATGGCTGCATGAGCTTGCTCTG
AGGAACATTTCATTGGAAGTGTTGAATTTTTATGTCCCATACTTAGAAAGCATTAACATGAG
AGATCTTGAGTTAATAGCCACGAACTGTCGAGCATTAACCTCTTTAAGGGTTAATGAATGTG
ATATTCTGGAATTAAGAGGTGTTCTGGATAAAGCTACAGAATTGGAAGAGTTTGGCGGTGGG
TCATTTCAATTTGTTAACAGTGATGAACATGCATTAGAAACTGATACATATCGAAGTGTTAA
ATTTCCTTCAAAGGTGATATCACTTATAGGACTCAATGACCTGAGCGAAACTGGGTTGCCTT
TTATACTTCCACGGGCTTGTAACCTGAAGAAATTGGATTTACAGTTTACATATTTGAGCACA
GAAAGTCATTGCGAGTTACTTTGTCTGTGCACTGGTCTTGAAGTTCTAGAGGTAAGGAATGG
AATTGGAGACAGAGGGTTAGAAGTTGTTGCAAACCACTGCAAAAAGTTAAGAAGACTGAGAG
TAGAGCCTGGGGAAGAAGAAGGTGAAGGTGGTGAGCAAAGTTCAGTCTCTCACAGAGGGCTT
TCTACCGTAGCTCAAGGCTGCCTCAATCTGGAGTTTATTGCTGTTTATGTTACAGATATAAG
CAACTTGGCATTGGAGACTGTTGGAGAATATTGCAAAAATCTTAGGGACTTTCGTTTAGTTT
TGTTGGATAAAGAAGAACAAATCACTGATTTACCACTTGATAATGGAGTCATGGCTTTGTTG
CGTGGGTGTAGCAAGTTGAACAGATTTGGCTTGTACTTAAGGCATGGGGGATTGACAGATAC
AGGACTTGGTTATATTGGGAAATACAGTGCAAATATAAGGTGGATGCTCTTGGGTTTTGTTG
GAGAATGTGACCAGGGTCTCCTTGAATTTTCAAAAGGATGCTTAAAACTGGAGAGACTTGAA
ATGAGAGGTTGTTGTTTCAGTGAATCTGCAATAGCGGCTGCAGTGCAGAACTTGAAATCATT
GAAGTATATCTGGGTGCAGGGATACAAAGGAACGACAACAGGTGAGAAACTTCTAGCTATGT
CACGACCATTTTGGAACATAGAGTTTACCCCACCTCTCAGAGAGACAGGGGATGAACTTGAT
GATATGGAAGAAGCAGTGATTGGACACCAACCAGCACAGATCTTGGCTTACTATTCTCTGGC
CGGAAGAAGAACAGATAATCCAAAATCTGTGATCCTTTTGTCGCCTCATTCTTGA

> COI1-like_MA_10436355g0010
ATGCTTGCATGTAAACAGGTTATGAATGTGACTGGAGATAGAGGTTTAGAAGTAGTTGTTGA
GAACTGTAAAAAATTAAGGCGACTTAGAGTGGAGCGTGGAGAAGATGAAGTTGGTTTGGAGG
ATGAGAAAGGCTCTGTTTCTCACAAAGGGCTCTCAGTTATGGCTCAAGGTTGTCCCAGTCTA
GAGTACATTGCTATGTATGCTTCAGATATGACTAACTCAACCTTAGAATCTGTTGGTAAATT
TTGCAAAAATCTGAGGGATTTTCTGCTAGTCTTGCTAGACAAGGAAGAACAAGTGACTGACC
TCCCACTGGACAATGGTGTCATGGCACTGCTTCTTGGGTGCCAAAAATTGAGGAGGTTTGGA
TTTTACCTAAGGCCTGGAGGATTGACGCACATAGGCCTTGGCTACATTGGAAAGTTTAGTAG
CAATGTGAGGTGGATGATTCTTGGTTATGTTGGAGAAACTAACATTGGACTTCTTGAGTTCT
CGAAGGGATGCCCAAATTTGGAGAAACTTGAATTAAGGGGTTGTTGCTTCAGTGAATATGCA
TTGTCCGTGACAGTTCTTAGCTTAAGGTCTCTAAAATATAGATGGGTTCAGGGTTACAATTC
AACGCCAACTGGAGTTGATCTTCTAGCTATGGAGTGTCCATTTTGGAACATAAAGTTTACTC
CAGCTTTCCATGTGACAGTGGATGGGTTTAATTTGGAAGCAGAAATTGTAGAGAATCCAGCA
CATATATTCTCTTATTATTCGCTAGCGGGAAGACGAACAGACCATCCAAATTCAGTAATTCC
GTTGACCTTATCATCATGGAATCGTCAGAATGTATATGGATATTAA

> COI1-like_MA_108477g0010
ATGGCCATGAAGCGGGCGGGATATGGATGCATATCAGAGGAGGCCCTGGAATGCGTCATGGG
TCAGCTGGAGGACCCGAGAGACCGGGGCTCGGTCTCTCTGGTCTGCAAGAAGTGGTACGACG
TGGATGCCTTCACGAGGAAGCATGTGACTGTGGCCTTCTGCTACTCGATACACGCCAGGGAC
CTCACCCGCAGGTTCACCAGGCTGGAGTCGCTCACGGTCAAGGGGAAGCCCAGGGCGGCCAT
GTATAATCTGCTTCCTGACGATTGGGGGGGTTATGCCAAGCCCTGGATAGACCAGATCTCGC

ACACCTGTCTCTGCCTCAAGACGCTCCATCTGCGCAGAATGATTGTTACCGATGATGATCTC
GCCACTCTCGTCAGGGGCCGCGGTCACATGCTGCAGGAGCTCAAGCTCGAGAAGTGCTCCGG
GTTCTCTACCAGGGGGCTCGAGGAAGTGGCGCACGGTTGCAGATCTCTTAAGACCTTGATGC
TGGACGAGAGTCAAATTGAAGAGGAAAGCGGGGATTGGCTACATGAGCTTGCTCTTAACAAT
TCTTCTTTGGAAGTGTTGAACTTCTACATGACAACAGTAGAAATGATCAATACCAGTGATCT
TGAGCTAATAGTAACAAACTGCCCCTCACTGACATCCTTAAAGGTTGGTGACTGTGATATAC
TGGATATGAGAGGTGTTCTGAGTAAGGGCACTGCATTGGAGGAGTTTGGTGGTGGTACATTT
AACACCAGTGAAGAGCATCCGACAGGGACCAATATGTCCCAGATGATTAAATTTCCTCCAAA
GTTGACATCATTGCTAGGACTAAACTTCATGATGGAGGCTGACATGCCTGCTATATTCCCAA
GAGCTTCTGCCCTTAAGAGATTGGATCTGCAGTACACATTTTTGAGCACAGAAAATCACTGT
CAGTTGGCAGGGCTCTGTCCTAATCTTGAAATTCTCGAGGTTAGAAATGTGATTGGAGACAA
AGGGTTAGAAGTTGTTGCAAATACTTGCAAAAAGCTGAAAAGACTTAGAGTGGAACGAGGAG
CAGATGACCCAACTTTGGAGGACGAACAAGGTTGGGTTTCTCACAAAGGGCTTTCCTCAGTA
GCTCAAGGCTGCCCCCTTCTTGAGTACATTGCTGTCTATGTTTCAGATATATGCAACTCAAC
ATTGGAGACTGTTGGTCAATGTTGCAAAAATCTCAAGGATTTCCGGTTGGTCTTGTTAGATA
AAGAAGAACACATCACTGATTTACCACTGGACAATGGAGTCATGGCTTTGCTACGTGGATGC
CAAAAACTGAGTAGGTTTGCATTTTATGTAAGGCCTGGAGGGTTGACGGATACAGGTCTTGC
TTATATTGGTGAGTACAGCACTAATGTCAGGTGGATGCTTCTAGGTTTTGCTGGTGAAACTG
ACCAAGGTATTCTCGAGTTTTCCAAGGGCTGCCCAAAGCTGGAAAGGCTAGAAATTAGAGGT
TGTTCTTTTAGTGAATCTGCATTGGCAGCTGCAGTGCTTCGTCTGAAATCGCTAAAGTACAT
ATGGGTTCAAGGATATAATGCAACTGTTACTGGCGCTAACCTTCTAGCTATGGCTCGACCTT
ATTGGAACATAGAGTTTTCTCCTGGTTTGCAATCGACAAAAGATGTGCTCGTTGAAGATATG
GCAGCAGAAAAAATGCAGGATCGGGTAGCACAACTTTTGGCCTACTATTCTCTTGCTGGAAA
TAGGACAGATCACCCAGAGTCTGTAATTCCCTTAGCCTCACCTTTCCGGAATTACCAACAAG
TAGCTGTTTTCTAA

> COI1-like_MA_15205g0010
ATGTTAATAGCCACAAACTGTCCAGCATTAACCTCTTTAAGGGTTAATGAATGTGATATTCT
GGATTTAAGAGGTGTTCTGGAGAAAGCTACAGAATTGGAAGATTTTGGTGGTGGGGCATTTG
ATAACATTGATGGACATGCATTAGAAACTGAATATCAAAGTGTTAAATTTCCTCCAAAGGTG
ATATCACTTGTAGGACTCAATTACATGAGTGACACTGGGTTGCCTTTCATTCTTCCTCTGGC
TTGTAACCTGAAGAAATTGGATTTACAGTTCACGTTTTTGAGCATGGAAAATCATTGCGAGT
TACTTCGTCTATGCACTAATCTTGAAGTTCTAGAGGTACGGAATGTGATTGGAGACAGAGGG
CTAGAAGTTGTTGCAAACCACTGCAAAAAGTTAAGAAGACTGAGAGTAGAGGCCGGGGAAGA
AGAAGATGAAGATGAAGAGCAACATTTAGTCTCTCACAGAGGGCTTTCTACAATAGCTCAAG
GCTGCCTCAATCTGGAGTTTATTGCTGTTCATGTAACAGATATAAGCAACTTGGCATTGGAG
GCTGTTGGAGAATACTGCAAAAATCTTAGGGACTTTCGTTTAGTTTTGTTGGATAAAGAAGA
ACAAATTACTGATCTGCCACTTGATAATGGAGTCATGGCTTTGTTGCGTGGGTGTAGCAAGT
TAAACAGATTTGGATTGTACTTAAGGTCTGGAGGGTTGACAGATACTGGACTTGGTTATATT
GGGAAATACAGTACAAATGTAAGCAGCTGCAGTGCTGAACTTGAAATCATTGAAGTATATCT
GGGTGCAAGGATACACGAGATCGGTCTCGGCATTCCTGCTGAACCGCTCTTCCGATCTCACG
AATCTGCAATAGCAGCTGCAGTGCTGAACTTGAAATCATTGAAGTATATCTGGGTGCAAGGA
TACCAAGGAACAGAAACAGGTAAGAAACTTTTGGCTATGTCACGACCATTTTGGAACATAGA
GTATATCCAACCTCTCCCAGGGACAGGGGATGAACTTAATGTTATGGAAGAAGAATTGATTG
GACACCAACCAGCACAGATCTTGGCATACTATTCTCTGGCCGGAAGAAGAACAGATAATCCA
AAGTCTGTGATCCTTTTATCACCTCATTCTTGA

> COI1-like_MA_263909g0010
ATGGGCAACGAAATCGCAGAAGAAGCCCTGGAGTTCGTGATGAGCTACGTCAACGACCCCAG
GGACCGAAGCGTGGTCTCTCAGGTGTGCAAACAGTGGTACAGGATCGATGCCCTGACGCGGA
AGCACGTGACCGTGGCCTTCTGCTACACCATAAGGCCCGCCGATCTCACTCGGCGCTTCAAA
CGGCTGGAGTCGCTGAAGCTGAAAGGGAAGCCCAGAGCCGACATGTTCAGACTCATCACGGA

GGACTGGGGCGCCTACGCCCGCCCCTGGATTACCGAGATCTCCTCCTCCTGCCTCTGCCTCA
AGACCGAGATCTCCTCCTCCTGCCTCTGCCTCAAGTCTCTCCATCTCAGGAGAATGGTCGTC
AAAGACGACGACCTCACTATGCTCGTCCGGAGCCACGGTCACATGCTGCAGGCTCTCAAGCT
CGAGCGGTGCTCGGGCTTCTCCACTCTTGGACTTCTGGAAATCGCTCGCTCTTGCAGGTAA

> COI1-like_MA_372199g0010
GTACGAAATGTGATTGGAGACAGAGGGTTAGAAGTTGTTGCGGACCACTGCAAAAAGTTAAG
AAGACTGAGAGTAGAGCCTGGGGAAGAAGAAGGTGAAGATGAGCAAAGTTTAGTCTCTCACA
GAGGGCTTTCTACCATAGCTCAAGGCTGCCTCAATCTGGAGTTTATTGCTGTTTATGTAACA
GATATCAGCAACTTGGCATTAGAGACTGTCGGAGAATATTGCAAAAACCTTAGGGACTTTCG
TTTAGTTTTGTTGGATAAAGAAGAGCAAATTACTGATCTACCTCTTGATAATGGAGTCATGG
CTTTGTTGCGCGGGTGTACCAAGTTAAACAGATTTGGATTGTACTTAAGGCCTGGAGGATTG
ACAGACACAGGGCTTGGTTATATTGGGAAATACAGTACAAATGTAAGGTGGATGCTGTTGGG
ATTTGTTGGAGAATGTGACCAGGGTCTCCTTGAACTTTCAAAGGGATGCTCAAAACTGGAGA
GGCTTGAAATGAGAGGTTGTTGTTTCACTGAATCTGCAATAGCTGCTGCAGTGCTGAACTTG
AAATCATTGAAGTATATCTGGGTGCAAGGATACAAAGGAACGGCAACAGGTGAGAAGCTTCT
GGCTATGTCACGACTGTTTTGGAACATAGAGTTTACCCCACCCCTCATAGTGACAAGAGATG
AACTTGATGATATGGAAAAAGAATTGAGTAGACTCAAACCGGCACAGATCTTGGCTTACTAT
TCTTTGGCTGGAAGAAGAACAGATAATCCAGAGTCTGTGATTCTTTTATCACCTCATTCTTG
A

> COI1-like_MA_437243g0010
GTCAAGCAAGCACTTCACAGAAGAAGAAGAATGCAGAAGCCGTGGAGGACATCAGCAAGCTG
TGCGATTCCGGACGAGGTTCTGGAATGCGTGATGGGGTACCTGGAGGACCCCCGGGATCGCA
GCGCGGTTTCCCTGGTCTGCAAGCGGTGTCTTCCGATCTCTGCGCACGGTTCTCCGGGCTTG
AGTCGCTGAAATTGAAAGGGAAGCCCAGGGCTGCCATGTTTAATTTGATTCCTCCGGACTGG
GGCGGATATGCGGGGCCGTGGATTAACGGGATTTCTGAGACATTTCTCTGCTTAAAGGCTCT
TCATTTGCGCAGAATGATCATTACGGACGAGGATCTCAGGGTTCTGTCTCGCGGCCGCGGCC
ACATTCTGCAAGTGCTTAAGCTGGAGAAGTGTTCGGGGTTTTCGACTGTCGGACTCCTCGAT
GTCGCACGCTCCTGCAGGTAA

> COI1-like_MA_63147g0010
ATGCAGAGGTCGTGGAGGATGTCTGTGAGCGATGCAATTCCCGACGAGGTTCTGGAGTGCGT
GATAGGGAACCTGGAGGATCCGCGCGATCGCAGCGCCGTTTCCCTGGTGTGCAAGAGGTGGT
ACCGCGTAGATGCTCTCACTCGCAAGCATGTTACCATCGCGTTCTGTTACACCGTAAGCCCT
TCGTATCTCAGCGCTCGGTTCCCACGGCTTGAGTTGCTGAAACTGAAGGGGAAGCCCAGGGC
TGCCATGTTTAATTTGATTCCACCAGACTGGGGCGGATATGCGGAGCCGTGGATTAACGAGA
TTTCCCAGACATTTCACTGCTTAAAGTCTCTTCATTTGCGCAGAATGATCGTTACCGATGAG
GATCTTAGGGTTCTGGGTTGCGGCCGCGGCAACATTCTTCAGGTGCTTAAGCTGGAGAAGTG
CTCCGGATTTTCAACTCTCGGGCTCCTCGAAATCGCACGCTCCTGCAGGTAA

> COI1-like_MA_63733g0010
ATGGAGAACCAGCGGGTGCTGATGATGGACAGAGGAATGCCAGAAGAAGCCCTGGAGTGCGT
GATGAGCTACGTGAACGACCCCCGGGATCGAAGTGTGGTCTCTCAGGTGTGCAAGCAGTGGT
ACATGATCGATGCCCTGACCCGGAAGCACGTGACCGTGGCCTTCTGCTACACAATCAAGCCC
GCTGATCTCACTCGACGCTTCAAGCGGTTGGAGTCGCTGAAGCTGAAGGGGAAGCCGAGGGC
CGCTATGTTCAACCTCATCCAGGAGGACTGGGGCGCCTACGCCCGCCCTTCAGGAGGACTGG
GGCGCCTACGCCCGCCCTTGGATTGA

> COI1-like_MA_646265g0010
ATGGCACATGAATGCTTTTGGCATGCATGTGCCCTCTATCTCTTGCTCCTCCATAAATGCCC
TTGCGGCATTGTGCACTCATTTTACTGTATGGAACTTCTGAAGGAGGGCATACTGCTGCAGC

TGTCTACCCTACTAGAAGAGGAAGTTATGATAGCTGGATCACAGTCCGAGCTACACATAGGT
AAAAACTACAGACCTAACTCCAAAATCATATCTCTTAAGGTCTTGCTATTAGAGGCCAGTAC
GATAAAAGATGAAGGTGGGGAATGGCTACATGAGCTTGCTCTGAGAAACTCTTCATTGGAAG
TGTTGAATTTTTATGAAACATTGTTAGACAGCATCGACATGAGAGATATCGAGTTGATAGCC
ACGAACTGTCGAGCATTAACCTCTTTAAGGGTTAATGAATGCTATATTATGGATTTAATAGG
TGTTCTGGAGAAAGCTACAGAATTGGAAGAGTTTGGTGGTGGGTCATTTGTTAACAGTGAAG
CACATGCATTAGAAATTGATAGATATCAACTTGTTAAATTTCCTTCAAAGGTGATATCACTT
ATAGGACTCAATTTTATGAGTGGGTTGCCGTTTATACTTCCACGGGCCTGTAACCTGAAGAA
ATTGGATTTACAGTTTACTCTTTTAAGCACAGAAAATCATTGCGAGTTACTTCGTCTCTGCA
CTAATCTTGAAGTTCTAGAGGTATGTCTCTGA

> COI1-like_MA_8264261g0010
GTTACGAATGTGATTGGAGATAGAGGTTTAGAAGTAGTTGTTGAGAACTGTAAAAAAGTAAG
GCGACTTAGAGTGGAGCGTGGAGAAGATGAAGTTGGTTTGGAGGATGAGCAAGGCTCTGTTT
CTCACAAAGGGCTTTCAGTTATAGCTCAAGGCTGTCCCAGTCTAGAGTACATTGCTATGTAT
GCTTCAGATATGACTAACTCAGCCTTAGAATTTGTTGGTAAATTTTGCAAAAATCTGAGGGA
TTTTCGGCTAGTCTTGCTAGACAAGGAAGAACAAGTGACTGACCTCCCACTGGACAATGGTG
TCATGGCACTACTGCTTGGGTGCCAAAAATTGAGGAGGTTTGGATTTTACCTAAGACCTGGA
GGATTGATGGACACAGGCCTTGGCTACATTGGAAAGTTTAGTAGCAATGTGAGGTGGATGAT
TCTGGGTTATGTTGGAGAAACTGACATTGAACTTCTTGAGTTCTCGAAGGGATGCCCAAATT
TGGAGAAACTTGAATTAAGGGGTTGTTGCTTCAGTGAATATGCATTGTCCGTGGTAGTTCTT
AGCTTGAGGTCTCTAAAATATATCTGGGTTTAG

> MYC2_AT1G32640
ATGACTGATTACCGGCTACAACCAACGATGAATCTTTGGACCACCGACGACAACGCTTCTAT
GATGGAAGCTTTCATGAGCTCTTCCGATATCTCAACTTTATGGCCTCCGGCGTCGACGACAA
CCACGACGGCGACGACTGAAACAACTCCGACGCCGGCGATGGAGATTCCGGCACAGGCGGGA
TTTAATCAAGAGACTCTTCAGCAACGTTTACAAGCTTTGATTGAAGGAACACACGAAGGTTG
GACCTACGCTATATTCTGGCAACCGTCGTATGATTTCTCCGGCGCCTCCGTGCTCGGATGGG
GAGATGGTTATTACAAAGGTGAAGAAGATAAAGCAAACCCGAGACGGAGATCGAGTTCGCCG
CCGTTTTCTACTCCGGCGGATCAGGAGTACAGGAAAAAAGTGTTGAGAGAGCTTAACTCGTT
GATCTCCGGTGGTGTTGCTCCGTCGGATGACGCTGTTGATGAGGAGGTGACGGATACGGAAT
GGTTTTTCTTGGTTTCGATGACGCAGAGCTTCGCTTGCGGTGCGGGATTAGCTGGTAAAGCG
TTTGCAACGGGTAACGCGGTTTGGGTTTCCGGGTCAGATCAATTATCCGGGTCGGGTTGTGA
ACGGGCTAAGCAAGGAGGAGTGTTTGGGATGCATACTATTGCGTGTATTCCTTCGGCGAACG
GAGTTGTGGAAGTCGGGTCAACGGAGCCGATCCGACAGAGTTCGGACCTTATTAACAAGGTT
CGAATTCTTTTCAATTTCGACGGCGGAGCTGGAGATTTATCGGGTCTTAATTGGAATCTTGA
CCCGGATCAAGGTGAGAACGACCCGTCTATGTGGATTAATGACCCGATTGGAACACCTGGAT
CTAACGAACCGGGTAACGGAGCTCCAAGTTCTAGCTCCCAGCTTTTTTCAAAGTCTATTCAG
TTTGAGAACGGTAGCTCAAGCACAATAACCGAAAACCCGAATCTGGATCCGACTCCGAGTCC
GGTTCATTCTCAGACCCAGAATCCGAAATTCAATAACACTTTCTCCCGAGAACTTAATTTTT
CGACGTCAAGTTCTACTTTAGTGAAACCAAGATCCGGCGAGATATTAAACTTCGGCGATGAA
GGTAAACGAAGCTCCGGAAACCCGGATCCAAGTTCTTATTCGGGTCAAACACAATTCGAAAA
CAAAAGAAAGAGGTCGATGGTTTTGAACGAAGATAAAGTTCTATCATTCGGAGATAAAACCG
CCGGAGAATCAGATCACTCCGATCTAGAAGCTTCCGTCGTGAAAGAAGTAGCAGTAGAGAAA
CGTCCAAAGAAACGAGGAAGAAAGCCAGCAAACGGTAGAGAAGAGCCACTAAACCACGTCGA
AGCAGAGAGACAAAGACGCGAGAAACTAAACCAAAGATTCTACGCGTTACGAGCGGTTGTAC
CAAACGTTTCAAAAATGGATAAAGCTTCGTTACTCGGTGACGCAATCGCTTACATCAACGAG
CTTAAATCCAAAGTAGTCAAAACAGAGTCAGAGAAACTCCAAATCAAGAACCAGCTCGAGGA
AGTGAAACTCGAGCTCGCCGGAAGAAAAGCGAGTGCTAGTGGAGGAGATATGTCGTCTTCGT
GTTCTTCGATTAAACCGGTGGGGATGGAGATTGAAGTGAAGATAATTGGTTGGGACGCAATG
ATTAGAGTTGAATCTAGTAAGAGGAATCATCCGGCGGCGAGGTTGATGTCGGCGTTGATGGA
TTTGGAGTTGGAAGTGAATCACGCGAGTATGTCGGTGGTTAACGATTTGATGATTCAACAAG
CGACGGTGAAGATGGGTTTTAGGATCTATACGCAAGAACAGCTCAGAGCAAGTTTGATTTCA
AAAATCGGTTAA

> MYC2-like_MA_15962g0010
ATGATGCAGTCTTTTGTTAATGAATGCGGGGTGCCTGGGAAGGCATTTTCCAGTGCCATGCC
TGTTTGGATTATCGGCTCAGAAAGGCTTCAGGGCTACAACTGTGACCGCGCTCGTCAGGCTC
AGCAATTTGGCATTCAGACCATGGTATGTATTCCGACACTTAACGGAGTTGTTGAGTTGGGT
TCCACGGATTTGATCCCCCAGAACTGGGATTTGATACAGAAGGCTAGAGATTCGTTTACTTT
TACGCTTCCGGACACTGCTTTGTGGGAAGAAAATCATACCCAAAATGATCCTGACCCGGCTC
TTTGGTTGACTGAACCCCCGGCTGAGCCTAAAACAGAAACTGAAAAGAAACAACAGCAGATA
CCCAATGCGGAAACTGAAGCTCTTCACAGCTTTTTTCCTCATGAATTAGGGTTTTCTGACCT
GGGGTTTCTGAATGGCGAGGGAGAAAACGCGTCACAGAAGCTTGTGGTTGAGGAGTGTGGGC
AGAAAGAAGATAAATCATCTCAGGGCTTTACTGGTTCTCAGGTTTCGTACCAACAGAACTGG
CAGGCTCAGACGACCTGCAAGACTGAAGTTGTGGATATTCCGGTGTTTCAACCTGGGAAAAG
GACTAATACTAATGGGATTACTCTGAGCTTTGAAAACCCCTACGGTGCTCAGGGTTTAGTGA
GTTGGGGGGATGAGAAGATGAAGAGGTCTGTGAGGAATGGGAATGAAGATGGTGGGAGTGGT
TTGTGTTTTTCTTCTGAAGTTACTGCTGTTTCTGCTTCTACTTCTGCTACTGCTGCTGCTGC
TGCTTTGCCTGTGAACGGTGCTGCTGGGGTTTCGTACCAACAGAACTGGCAGGCTCAGACGA
CCTGCAAGACTGAAGTTGTGGATATTCCGGTGTTTCAACCTGGGAAAAGGACTAATACTAAT
GGGATTACTCTGAGCTTTGAAAACCCCTACGGTGCTCAGGGTTTAGTGAGTTGGGGGGATGA
GAAGATGAAGAGGTCTGTGAGGAATGGGAATGAAGATGGTGGGAGTGGTTTGTGTTTTTCTT
CTGAAGTTACTGCTGTTTCTGCTTCTACTTCTGCTACTGCTGCTGCTGCTGCTTTGCCTGTG

AACGGTGCTGCTGGGGTGAGATCTAGTGTTGAATCGGAGCATTCAGATATAGAGGCGTCTTT
TAAAGAGGCCGAATGCAGCCAGGCCATTGTTGAAAGGAGGCCTCGGAAACGGGGAAGGAAGC
CTGCCAATGGTAGAGAAGAACCTCTGAATCATGTAGAAGCTGAAAGGCAGAGGCGAGAAAAG
TTGAACCAGAGGTTTTATGCACTCCGTGCTGTGGTTCCCAATGTGTCCAAGATGGATAAGGC
TTCTCTGTTGGGTGATGCCATTTCTTACATTAATGAGCTCCGAAACAAGGTGCAGGATTCCG
ATTCTCATAAAAAGGACTTGCAGGCTCAACTCGAGGCCTTGAAGAAGGAATTGGTAGCCAGA
GAATCCGTTGCTTCTGGATTTTCTGGCAGTAATTTTGGTTTGCTAAAAAACCCATCTGCTGC
TGATCCTTCAAACCTTGATGTCAAAGGCTTTGGTTTGAAAAACCAGTGTCCTAATATTGAGC
TTGAAGTACGAATCCTTGGTCGAGAGGCCATGGTCAGAGTTCAGTGTCCCAAGCAGAACCAT
CCTGTTGCAAGATTGATGGTTGCATTTAAAGAGCTTGAACTTGAAGTCCATCATGCCAGTGT
ATCCACAGTGAAGGAGTTGATGATTCAAACGGTTATTCTGAATATGACAGGTATTGTATATA
CGCAGGAACAACTAAATGCTGCATTATTAAGGAAAGTAGCAGATCCTGGCCTTAGATAG

> MYC2-like_MA_5838g0020
ATGGTGGAGGCATTCATGGCGTCGTCTTCGTCGTCGTCGTTTTTGGCGGAGTATCCGCCATG
GAGCAACGCAGGCGGGGACCCGAACGGTTCATTCAATCAGGAGACACTGCAGCAGAGGCTGC
AGCTCTTGATCGACGGAGCGCGCGAGAGGTGGACATACGGGATTTTCTGGCAGTGGTCGTAT
GAGGCCGGCGGTGCCGTGGTCTTCTTCTGGGGCGACGGCTACTTCAAGGGCGCCCTCGAGGA
CGAGAGGACGAACACCGTGGCTGTCGCCAAGCGGACAACGAAGGAGAACGCGGCGCACCAGG
AGCTCAGGAAGAAGGTGCTCCGCGAGCTGCACGAGCTCATCGACGGGGCCTCCGATCCCGCC
GACGAGGAGGTGACGGACGTGGAGTGGTTTTATCTGGTCTCCATGACGCATTCGTTCACAGG
GGTGGAGGGGGTTCCGGGGCACGCGTTCATGTCCAGCGCGCCCGTGTGGCTGTCTGGGTCGC
TGAAATTGGAGTCGTTCGGATGCCAGAGGGCCAGGCAGGCTTCTCAGTTCGGGATTCAGACC
ATGGTATGTATTCCAACTCCTAATGGCGTTGTGGAATTGGGTTCTATGGATCTGGTCTGTGA
GAACTGGGGTCTGCTGCAGCAGGCCAAGAGCTCATTTACTTTTTCCTCTAGTTTCTGGGAAG
ATAACGGGAATGGTAATATTAATCATAACAATAACCATAATAATAATTATGGTACTAATAAC
CAGTCGTTATGGAATCCGGGGTCTCCATTCTTGACCCAGGAATCGATCTTGGGTGACTTGAG
TTTTCTCAATAACGAGGAAAGCCAAAACCGGAATTCGTCTGCTCAAAAATCCTTGTCCATCT
TGGAAGAAAACCGAAACCCTTTACCCCCTTTTACTGTTCAAAAGCAAGTAGCCCTGGAGGAA
AACCGGATTCCGCCCTCTTTTCCTGCTCAGAAATCTGCCGTCCTTGACGAGAGAACCTCCTT
ACCTCTTGTTCAGAAAACTGGCATCTCCGAAGGAGCTCATAATCCCCTGCCGTTCCTTTCAA
AGCCACCTCCTGGAGGCGGCTTTGATGAGAAGTCTAATACTTTGCCATATTCTGCTGTTCAA
AGGCCTGCTATCATTGATGAGAACTGCAATTCTTTGCTTTCTCAGAAATCCAGCATCTGCAG
TGAGATTCTTAATTCTTACCCATATGTGACCGGTCCAAAATCTGTTGATTTCAGCGAAACTT
GTAATTCTTATCCAGTTCCAGCTCTTCAGAAAACTGGCATTGAAAACAGTAATCAGAATTTA
CTACAATCCGCTGCTGTTCTGAAACCTAACATAGCGGACCAAATTGGTAATCCGAATTCAAA
CCCAAATCCGCTGCTTTTTTCTGTTCAGAAAACTTGTACTATTGATCAAAGCAAGGGATTCA
TCAAAGATCTCCTGATAGAGGACGACAAGCCCAAGCCCTTGTTGGATTTCCAGAGACACACT
CTGAGCTTTGCAAATGGATACTCGCAGAATATGGTGGAGGAGAAGATGGTGAAACCTCTCAG
TATTGATGATGAAAAGCCCAAGTCCTTGCCTACAATTTCCTCTGGTGCTGTCTTTGGTGGAG
TTCGATCTAGTATTGAATCTGACCATTCTGATGTAGAAGCTGCGTCCTTTAAGGAAGCAAGC
CAAGCTGTCATTGAGAAAAAGCCCCGGAAAAGAGGAAGAAAGCCTGCAAATGGTAGAGAAGA
GCCTCTGAATCATGTTGAAGCTGAGCGTCAACGGCGCGAGAAACTGAATCAGAGATTCTATG
CACTTCGCGCTGTAGTACCAAATGTGTCAAAGATGGATAAAGCTTCATTGCTTGGGGATGCT
GTGTCCTATATTAATGAACTTCAAAGCAGAGTTCAAGACATTGAATCTGAGAAAAAGGAGCT
TCAAGCCCAAATAGAAGCTACCAAGAAGGAATCCTCGTCTTCTCACTCTGCTTTCTCTGGTA
CCAATTTGGGATTCATCAAGGACCAGTCCGGTTCATCTCAAAAGCCTGATGTCAAACGATTT
GGCACAAAGGAATGCTCTGCACTAGATCTGGAAGTTCGAATCCTTGGTCCAGATGCCATGAT
CAGAATTCAGTCAGCTAAGAAGAATCATCCTGCAGCCAGATTGATGACATCACTGCAAGACC
TAGAGCTTGAAGTTCATCATGCCAGTGTCTCAACAGTCGACGAGTTAATGCTTCAAAATGTG
ATTGTCAAGTTACCAAGTAGTTTATATACTGAAGAACAACTCAATGCCATCCTGTTGAAGAA
ATTATCAGATCCAAAATTTAAGTAA

> MYC2-like_MA_10435905g0020

ATGGATACTTTAATGGCGTCATCAGTGGTAGATCAGAGGTTCAGCCAGGAAACATTGCAGCA
GCGCCTGCAGACTCTAGTAGAGACGGCCTCAATAGTGTGGACTTATGCGATTTTCTGGCAAG
TTTCTTATGAATCTAGCGGTGCCATTCAGCTATGCTGGGGAGACGGCTATTACAAGGGGTCG
AGGAATACGGAAGAGGATGAGCGGCTTAGGATGCGCAGCCGTTTGACCGTGAGCCCCGCCGA
CCAGGAGCTGAGGAAAAAGGTATTACGCGATTTGCATTCTATGATTAGCGGGAGCGATGAAG
GAAACCAGCAGGATAATAGTAGTGTTAGCGTAGATGAAGAAGTTACTGATGCTGAATGGTTT
TATTTAATTTCGATGATGCAATCCTTTTTATCTGGGTTTGGGGTACCCGGTACGGCATTTTC
TACCGGAGCTCCTGTTTGGATCGTCGGAGCGGAGAGACTGCGGGTATCGACATGCGACAGGG
CAAGGCAAGCTCATGACCTTGGGATTCAAACACTTGTCTGTGTTCCGATTCAGGGCGGCGTG
GTTGAATTTGGATCGACGGAAGACATTGTTGAAAACTGGTTGTTTCTGGAGCAGGTCAACCG
TTCGTTTAAATATAACCTCAACCAAACGCATGATAACCTGTTTCAAGTCCAGTCTTTATGGC
CAGAGGAAACTTTGAGTGTTAAAAGCAGCAATACTATGCAATCCGCGCCATGCATCGAGCCT
GTGAACAATGCTGAAATCCAGTCCCTGAACTCTGCTTTAGCTCGGGAATTACCAGTGACAGG
CAAACAAAAGGCATCTGTGTTCGCTGAACAGAGTTCTTTAGTGGTTAAAGACGACAAATCTT
TGCTGCATCCCCTAACTCAGCAGACCGAAGCCCTTGAAGCGCCGGCCATCCGTATTCCAGAA
ACAGTAAACGGTACAGAGCCACAAACCCGCGCATTGGGCTTTAAAGGCAGTGAAAAGAATGT
GATAAAGCCATCAATAAAAGAGGATACCATTGGTTTACTGTCTAATCCGCCTGGAATTGTCA
TAGGAGGCCTGCGTTCTAGCATTGAATCAGAGCTGTCTGATGCAGAACCCTCTGCCTCAATT
AAAGATTCAACTTCTGCTGTAGTCGAGAGAAAACCACGGAAACGTGGGAGAAAGCCTGCAAA
TGGCCGCGAAGAGCCTCTGAATCATGTTGAGGCTGAGCGGCAAAGGCGTGAGAAATTGAACC
AGAAATTTTATGAGCTTCGTGCTGTGGTTCCCAATGTGTCGAAGATGGACAAAGCTTCTCTG
CTCGGCGATGCCGCTGCTTATATCAAAGACCTCTGTTCCAAACAGCAGGATTTGGAATCCGA
GAGGGTTGAATTGCAGGATCAAATTGAGTCTGTAAAGAAGGAATTATTGATGAATTCTTTGA
AGTTGGCAGCTAAAGAAGCAACAGATCTTTCAAGTATTGACCTTAAAGGTTTTAGCCAGGGG
AAGTTCCCCGGCTTGAATTCAGAAGTTCGCATTCTTGGCCGAGAGGCGATAATAAGAATTCA
ATGCACTAAACATAATCATCCTGTTGCGAGACTGATGACAGCACTGCAAGAACTTGATTTGG
AAGTCCTCCATGCGAGTATTTCTACTGTGAAGGATTCGTTAATTATCCAGACAGTCATTGTT
AAAATGACCAGAGGTTTGTACACGGAAGAACAACTTCATGCGCTGCTTTAGCAGGTAATGGA
CTGCTCAAGGATTGTTTTAGAATTTGCATTGGTCTCAGCATAA

> MYC2-like_MA_9725g0010

ATGACAACGGAGGAGAGAGCAACACAACATGAGCTCAGGAAGAAGGTGCTCCACGAGCTCAT
CAATGGGGCCTCTAATCCTGCGGTCGATGAGGAGGTGACGGATGTGGAGTGGTTTTATCTAG
TCTCCATGATGCATTCATTCATAGGGGCCAAGGTGGTTTCGAGCCATGCAGTCATGTACAAT
GCACCTATATGGCTCTCCGGGTCACCGAAATTGGAGTCATTTAGATGTTAG

> MYC2-like_MA_140086g0010

ATGGGCAACGAAGGCGCGGGATCGATGCGGCTGTGGGGCGATGATAATAATGTCATGATCGA
GGCCTTCATGGGGAACCTGGATTACTCCTACTCGTTCCCCTGGAATGGCATCGACGCCAATC
CCTCCGCTCTACCCTCGCCCGCCCACTTCGCATCGTCTGCCGCCAGCGTTGCTATTGCTACG
CCCTTCAATCAGGACACGCTGCAGCAACGACTGCTGGCGCTTGTGGAGGGCGCTACCGAGAG
CTGGACG

> JAZ10_AT5G13220
ATGTCGAAAGCTACCATAGAACTCGATTTCCTCGGACTTGAGAAGAAACAAACCAACAACGC
TCCTAAGCCTAAGTTCCAGAAATTTCTCGATCGCCGTCGTAGTTTCCGAGATATTCAAGGTG
CGATTTCGAAAATCGATCCGGAGATTATCAAATCGCTGTTAGCTTCCACTGGAAACAATTCC
GATTCATCGGCTAAATCTCGTTCGGTTCCGTCTACTCCGAGGGAAGATCAGCCTCAGATCCC
GATTTCTCCGGTCCACGCGTCTCTCGCCAGGTCTAGTACCGAACTCGTTTCGGGAACTGTTC
CTATGACGATTTTCTACAATGGAAGTGTTTCAGTTTTCCAAGTGTCTCGTAACAAAGCTGGT
GAAATTATGAAGGTCGCTAATGAAGCAGCATCTAAGAAAGACGAGTCGTCGATGGAGACAGA
TCTTTCGGTAATTCTTCCGACCACTCTAAGACCAAAGCTCTTTGGCCAGAATCTAGAAGGAG
ATCTTCCCATCGCAAGGAGAAAGTCACTGCAACGTTTTCTCGAGAAGCGCAAGGAGAGATTA
GTATCAACATCTCCTTACTATCCGACATCGGCCTAA

> JAZ10-like_MA_10229741g0010
ATGTACCGCGCAGCTATGGACTCCATGGGCGTCGATACTAAGGGATCCAGTATTAACCGAGC
CCCTGAATCTGGTCCGGCGGATCAACTCAAAAACGATAGAGAAAGAGCCTGCATGAACAATC
TATCGTGGATAAAACCCCAAATGATGCGACAAGTGCTTTCGTTTAGAGATGAAGCAAGAAAC
GCGTCATGCCATGGAGTATCTCAGGTTCGTATGGATTCCTGCGCTCCAGGGCTAATGGGATT
ATTTCGTCAGCCAATAGGTTTTGTGGCTTCCATTTCTCCTGCTGCAAACCACGAGACCTCGG
CTTGCAAATATACTCATAATACCGCGGCAGCAGGTAGTGTGCAGTCACAACGATGGCCTTTC
CTCTCCCATGGATCTGTCCAGACAAATGGGACAATTTCCAGGCCGGCGTCTGCAACGGCTAC
AGAAGGCAAGACAACGACTGCCCCGTTGACGATCTTCTATAACGGCACGGTCAGCGTTTACG
ATGTCCCTGCGCACAAGGCTAAAGCAATCATGATGCTGGCCACAACTGCAATCTCTAATTGT
AAGGCAACTTCTCCTACCTCTACCATTACGACTACATCCGGAGCTTCAGCCAGTAAACTGAT
CTCCATGGCAGTACCAACCGAGACATTTACAGGAGCGGCCGCGCAGCAACAAACAGCTTGCA
AGCCGAATGCTGGACTGCCAATTGCGAGGAAACAATCGCTGCAACGTTTTCTCGAGAAGCGG
AAAGAGAGACTGAACATCGCCAGTCCGTACGCGATGAAAACCTCCGAATCCAACGAGGAATG
CGCCAGTGCACCCATCGTTTTTCCGACGGTGTAA

> JAZ10-like_MA_8757791g0010
ATGATGCGACAAGTGCTTTCGTTTAGAGATGAAGCAAGAAACGCGTCATGCCATGGAGTATC
TCAGGTTCGTATGGATTCCTGCGCTCCAGGGCTAATGGGATTATTTCGTCAGCCAATAGGTT
TTGCGGCTTTCATTTCTCCTGCTGCAAACCACGAGACCCCGGCTTGCAAATATACTCAGAAT
ACCGCGGCAGCAGGTAGTGTGCAGTCACAACGATGGCCTTTCCTCTCCCATGGATCTGTCCA
GACAAATGGGACAATTTCCAGGCCAGCGTCTGCAACGGCTACAGCAGGCAAGACAACGACTG
CCCCGTTGACGATCTTCTATAACGGCACGGTCAGCGTTTACGATGTCCCTGCGCACAAGGCT
AAAGCAATCATGATGCTGGCCACAACTGCAATCTCTAATTGTAAGGCAACTTCTCCTACCTC
TACCATTACGACTACATCCGGAGCTTCAGCCAGTAAACTGATCTCCATGGCAGTACCAACCG
AGACATTTACAGGAGCGGCCGCGCAGCAACAAACAGCTAACAAGCCGAATGCTGGACTGCCA
ATTGCGAGGAAACAATCGCTGCAACGTTTTCTCGAGAAGCGGAAAGAGAGACTGAACATCGC
CAGTCCTTACGCGATGAAAACCTCCGAATCCAACGAGGAATGCGCCAGTGCACCCATCGTTT
TTCCGACGGTGTAA

> JAZ10-like_MA_15372g0010
ATGCAAAGCATCTGGATATGTACAATCCCGTGCGAAGGTGGGAACTGGAACCAAGTGCCTGA
CCCTGCCTATGCTCGTCTTCGCCTCCTTTACCTTCAGCGCAGAGGGAGCCTTGATCTTGCTT
GGGCCTCGACCTCAAGGGCTCCAGTATTAACCGAAACCCTAAATCTGAACCTGCGGATCAGT
TCAACAATGACAAGGACAGAGAATCAGTCGTGGATAGAGCCCCAGGTGATTCAGCAAGTCCT
CTCGTTTACGGATAGGGCGAAAGGCGAGTCGTGCATCTATAGAGGGTCTCAGGTGCTGTTGA
ATTCCTCCGCTCCGGGCTTAATGGAGTTATTTCCTAAAGAAACAGTATTTGCAGCTTCCAGT
TCTGCTGCCACAAAGCACAAGAGCCCTTATTGCAAGAGTACTCTTCAAGAGACCGCAGTTCC
AGCTAGTGTGCAGTCACACCGATGGCCATTCCTCTCCCACCGATCTGTCGAGACAACTGCTA
CGATTTCGAGGACGGCCTCTGCCCCGTTGACAATCTTTTATAACGGGAAGGTCAGCGTTCAT

GATGTCCCTGCTCACAAGGCTGAAGCGATCATGATGTTGGCCACCACTTCAATTTCTAATCG
CCAGGCAAGTTCTTCTCCTCCCACTACCTATGGGCCTTCAAGCGGTACATTCAGGTCCACGG
AAATACCCGCAGAGCCATTCGAGGCCGCATCTGCGCAGCCAGAATCGACTCGAAAATTGATA
ATCGGAATGCCCGTTGCGAGGAAACAATCGCTGCAGCGTTTTATTGAGAAGCGAAAAGAGAG
ACTGAACAGCGCAAGCCCTAATTACAAAATGAATCCGACGACATCAAAACCCGTCGCGTGCG
TCGAGGGATGCGGCGGTGCTTCCCTTATTTCTCCGATGGTGTAA

>AOC1_AT3G25760
ATGGCTTCTTCTACAATCTCTCTCCAATCTATCTCCATGACAACTCTCAACAATCTCTCATA
TAGTAAACAATTTCATCGAAGCTCTCTTCTTGGTTTCTCTAAATCCTTCCAAAATTTTGGTA
TCTCATCTAACGGTCCAGGTTCCTCCTCTCCGACAAGTTTCACGCCCAAGAAGAAACTCACT
CCTACTCGAGCTCTCTCTCAGAACTTGGGAAATACCGAAAACCCCAGACCAAGCAAAGTTCA
AGAACTGAGCGTGTACGAAATCAATGATTTAGATCGACACAGCCCAAAAATTCTTAAAAACG
CATTCAGCTTTAGGTTTGGTCTCGGAGATCTCGTCCCATTCACAAACAAACTCTACACCGGC
GATCTCAAGAAACGCGTGGGAATCACGGCTGGTCTCTGCGTTGTGATCGAACACGTCCCTGA
GAAGAACGGTGATAGATTCGAAGCTACGTACAGCTTCTACTTCGGAGACTATGGCCACTTGT
CCGTACAAGGACCATACTTGACTTACGAGGATTCGTTCCTTGCCATCACTGGTGGCGCTGGA
ATCTTTGAAGGTGCCTACGGACAGGTTAAGCTTCAACAGCTTGTGTATCCGACAAAACTGTT
CTACACTTTTTATCTTAAAGGATTGGCTAATGATTTGCCACTGGAGCTCATAGGAACTCCGG
TACCACCGTCTAAGGACGTGGAGCCAGCACCGGAAGCTAAGGCGCTTAAGCCCAGTGGAGTT
GTAAGCAACTTTACAAATTAG

> ACO1-like_MA_137604g0010
TGGCAGCGTCTGCTCAAAGCCGAATACAATTTCACAGCTCCTCTCTGTTGAAGCCAGAGGCA
TCAATTCGACATCCTCAGTACAGATACTGGAAATGCTATTTCACAGCTCCATACAATTTCAC
AGCTCCTCTCTGTTGAAGCCAGAGGCATCAATTCGACATCGGGCGAAGGCCATCTCTTCTTC
TTCTTCTGCGCAAGGGCCTCCAAGCGGCTCTGTTCAGGAGTTGCACGTGTATGAGATAAATG
AATTGGACAGAGGGAGTCCTGCTTACCTTCGTCTCAGTCAGAAGCCCGTCAATCATCTGGGA
GATCTAGTACCATTTAGCAATAAGCTGTACACTGGCAATCTACAGAAGCGTATAGGAATAAC
AGCTGGGATCTGTATTCTCATCCAACATTTTCCTGAGAAGAAAGGAGACCGTTATGAAGCAA
TCTACAGTTTTTACTTTGGAGATTATGGCCACATTGCTGTGCAGGGACCTTATCTGACATTC
GAGGACGAGGAATCATACCTTGCAATCACTGGGGGTTCTGGCATATTCAACGGAGTTCGTGG
GCAAGTGATGTTAAAGCAGCTTGTCTTTCCTTTCAAGATATTCTACACATTTTACTTAGAGG
GCATTCCAAGCCTGCCGGAAGATCTGCTGGGTGAGCCTGTGGCACCGACACCTGATGTGGAG
CCATCTCCCGCTGCAAAGAATAAAGAGCCTCATGCCTGTGTGCCCAATTATACACAGTAA

> ACO1-like_MA_56386g0010
ATGGCTGCTACTGCCACTGCCACCTCGATCTCATTAAAATCATCCTTGGGAAACAACAAAAG
CCTGCATCAATTGGTTTGTTCAAACTCAGAGGGCAACAATACTAATTCTATAGGCTTCAAAC
CTGCTACTTGTTTTAAGCTGTTAAAGGGTGAGATTTTTGGGGTACAGTTGGCTACTGAGAGG
CAAATCCAGTTTGACAGCTCCTCTCTGGTGCGAGTGAGGGCAGGGGCAGAGACATCGATATC
AATTCGACGTCGAGTTAAGGCCAACGCTTCTTCTGCTCAAGGCCCCCCAAGCGGCTCTGTGC
AGGAGTTGTATGTGTATGAGATGAATGAGTTGGACAGAGGAAGCCCTGCTTACCTTCGTCTG
AGTCAGAAGCCCGTCAATCATCTGGGAGATATAGTGCCATTTAGCAATAAGCTGTACAGTGG
CAATCTGCAGAAACGTCTAGGTATAACAGCTGGGATCTGTATTCTCATCCAGCATTTTCCTG
AGAAGAACGGAGACCGTTATGAAGCAATCTACAGCTTTTACTTTGGAGATTATGGCCACATT
TCTGTGCAGGGGCCTTATCTGACATACGAGGAATCATACCTTACAATTACTGGGGGTTCTGG
CATATTCAGTGGAGTTCATGGGCAAGTGAAGTTAAAGCAGATTGTCTTTCCTTTCAAGTTAT
TCTACACTTTTTACTTGGAGGGCATTCCAAGCCTGCCCGAAGATCTGCTGGGTGAGCCTGTA
TCCCCAACACCGGATGTGGAGCCATCTCCCTCTGCAAAGAATGAAGAACCTCATGCCTGCCT
GCCCAACTATACTATGTAG

Table S1 Primers used for qPCR experiments in this study

Gene ID	Given Name	Forward (5'-3')	Reverse (5'-3')	Efficiency
MA_108477g0010	*PaCOI1*	GCAACTGTTACTGGCGCTAACCTTC	GCCATATCTTCAACGAGCACATC	2,57
MA_5838g0020	*PaMYC2*	AATACTTTGCCATATTCTGCTGTTC	GGATAAGAATTACAAGTTTCGCTGA	2,353
MA_6326g0010	*PaJAZ3*	AAGGGAGAGCAAGCAGCAGGAACAAC	AGAGGCTCCGACAACAGGCAAGAAAG	2,32
MA_10229741g0010	*PaJAZ10*	TGGCTTCCATTTCTCCTGCTGCAA C	CCGGCCTGGAAATTGTCCCATTTGTC	2,942
MA_56386g0010	P*aAOC1*	CTCCTCTCTGGTGCGAGTGA	GGGCTTCCTCTGTCCAACTC	2,466
MA_50378g0010	*PaEIF4A1*	TTGGTCGGAGTGGACGATTTGG	GTCCTGCAGCATTCTCTCGTCA	2,041
MA_13020g0010	*PaUBC28*	GGATCTCTGTTAACCGCGTCGTTG	AGGATCCGCTTAGACGCCATT	1,989
